# Integrative analysis reveals the functional implications and clinical relevance of pyroptosis in low-grade glioma

**DOI:** 10.1038/s41598-022-08619-w

**Published:** 2022-03-16

**Authors:** Lin Shen, Yanyan Li, Na Li, Yajie Zhao, Qin Zhou, Liangfang Shen, Zhanzhan Li

**Affiliations:** 1 Department of Oncology, Xiangya Hospital, Central South University, No. 87, Xiangya Road, Kaifu District, Changsha, 410008 Hunan People’s Republic of China; 2 Department of Nursing, Xiangya Hospital, Central South University, Changsha, 410008 Hunan People’s Republic of China; 3 Department of Nuclear Medicine, Xiangya Hospital, Central South University, Changsha, Hunan 410008 People’s Republic of China; 4 National Clinical Research Center for Geriatric Disorders, Xiangya Hospital, Central South University, Changsha, 410008 Hunan People’s Republic of China

**Keywords:** CNS cancer, Gene regulatory networks, Prognostic markers

## Abstract

Using the Chinese Glioma Genome Atlas (training dataset) and The Cancer Genome Atlas (validation dataset), we found that low-grade gliomas can be divided into two molecular subclasses based on 30 pyroptosis genes. Cluster 1 presented higher immune cell and immune function scores and poorer prognosis than Cluster 2. We established a prognostic model based on 10 pyroptosis genes; the model could predict overall survival in glioma and was well validated in an independent dataset. The high-risk group had relatively higher immune cell and immune function scores and lower DNA methylation levels in pyroptosis genes than the low-risk group. There were no marked differences in pyroptosis gene alterations between the high- and low-risk groups. The competing endogenous RNA (ceRNA) regulatory network uncovered the lncRNA–miRNA–mRNA regulation patterns of the different risk groups in low-grade glioma. Five pairs of target genes and drugs were identified. In vitro, CASP8 silencing inhibited the migration and invasion of glioma cells. The expression of pyroptosis genes can reflect the molecular biological and clinical features of low-grade glioma subclasses. The developed prognostic model can predict overall survival and distinguish molecular alterations in patients. Our integrated analyses could provide valuable guidelines for improving risk management and therapy for low-grade glioma patients.

## Introduction

Gliomas are the most common type of primary tumour in the central nervous system and one of the most devastating tumours^1^. According to the World Health Organization, glioma can be divided into low-grade and high-grade glioma. Low-grade glioma (LGG) is a more common primary neuroepithelial intracranial tumour; it includes WHO grade II–III gliomas and has a high incidence^2^. Although its pathological grade is lower, recurrence and malignant conversion still occur after standardized treatment, and the survival prognosis is poor^3,4^. Factors that influence survival in LGG may include phenotypes and administration of appropriate treatment^5^. The primary reason for the poor survival prognosis may be that the molecular mechanism is still not fully understood. Therefore, it is of great theoretical and practical significance to explore and study the underlying mechanisms of glioma, identify potential therapeutic targets, and apply them to clinical practice.

Modes of programmed cell death include apoptosis, ferroptosis, necroptosis, pyroptosis, and autophagy^6^. Pyroptosis was first described as caspase-dependent cell death in macrophages by Cookson^7^. Pyroptosis is mainly mediated by the activation of various caspases, including caspase-1, through the inflammasome; these caspases cleave the amino- and carboxy-terminal linkers of gasdermin D (GSDMS), resulting in perforation of the cell membrane and causing cell death^8^. Pyroptosis is an important response of the innate immune system to pathogens, which is closely related to the inflammasome, and inflammasome activation can promote pyroptosis^9^.

In the present study, we first outlined the molecular subtypes of low-grade glioma based on pyroptosis genes and described the clinical and molecular characteristics and immune status of each subclass. Then, we established a prognostic model of pyroptosis genes in the CGGA training cohort and validated this model in the TCGA validation cohort. Furthermore, we explored immune infiltration, somatic copy number alteration, and DNA methylation and constructed a lncRNA–miRNA–mRNA regulatory network. Finally, we explored the correlations of small molecule drugs with the identified prognostic genes. Our integrated analyses uncovered the biological mechanisms and function of pyroptosis in the occurrence and progression of low-grade glioma.

## Materials and methods

### Data source

The data used in this study consisted of two public datasets. The 630 low-grade glioma samples with transcriptional data and clinical data of the corresponding patients were obtained from the CGGA (http://www.cgga.org.cn/). The validation cohort containing 529 samples was obtained from the TCGA database (https://portal.gdc.cancer.gov/) and included gene expression profile, copy number alteration, methylation, and clinical data. Immune cell and immunophenoscore data were accessed from the TCIA database (https://tcia.at/home). Drug response data for 1000 cancer cell lines were downloaded from the Genomics of Drug Sensitivity in Cancer (GDSC) database (https://www.cancerrxgene.org/downloads). Thirty-three pyroptosis genes were obtained from previous reviews and studies, and these genes and their Entrez IDs are listed in Table S1^10–13^.

### Identification of molecular subclasses and gene set variation analysis

We identified the molecular subclasses using the consensus clustering method, which can determine the optimal cluster number via a cumulative distribution function. We also used a t-distributed stochastic neighbour embedding-based approach to validate the clustering consensus. We used the GSVA R package to determine the potential pathway enrichment by calculating the enrichment scores of each sample.

### Development and validation of the prognostic model

We established a prognostic model of overall survival in the CGGA training dataset using pyroptosis genes. LASSO regression was performed to identify the potential genes in the prognostic signature, and a Cox regression model was developed. Then, we calculated the risk score of each sample using the following equation: risk score = coef1*gene_1_ expression + coef_2_* gene_2_ expression + …coef_n_*gene_n_ expression. The patients in the training and validation cohorts were classified into the high-risk (risk score > median risk score) and low-risk (risk score ≤ median risk score) groups. We calculated the 1-year, 2-year, and 3-year areas under the curves (AUCs) to evaluate the predictive ability of the prognostic model. Finally, we developed a quantized assignment operator to calculate the 1-year, 3-year, and 5-year survival probabilities of individual patients based on the prognostic model, which were evaluated by consensus calibration analysis.

### Functional enrichment analysis and estimation of tumour stem cell-like properties and immune infiltration

Gene Ontology and KEGG pathway analyses were carried out using the TCGA and CGGA datasets separately^14,15^. To calculate the enrichment scores of stem cell-like properties (RNAss and DNAss) and the TME (stromal score, immune score, and ESTIMATE score), we carried out single-sample gene set enrichment analysis in the TCGA dataset. In addition, the immune-related cell and function scores were calculated for each sample (downloaded from https://www.gsea-msigdb.org/).

### Somatic copy number alteration, mutation, and DNA methylation analysis

We further compared the copy number alteration frequencies and DNA methylation levels between the high- and low-risk groups using the “limma” R package.

### Construction of the ceRNA network and analysis of drug sensitivity

To investigate the potential lncRNA–miRNA–mRNA regulatory network, we identified the differentially expressed lncRNAs, miRNAs, and mRNAs between the high- and low-risk groups. Then, we constructed the lncRNA–miRNA–mRNA regulatory network using Cytoscape (version 3.8.2). Based on the correlation coefficient (|R| > 0.25, P < 0.05), we identified the potential small molecule compounds related to the pyroptosis genes included in the prognostic model.

### In vitro experimental verification

We further performed quantitative polymerase chain reaction, Western blot analysis, migration assays, invasion assays, and clonogenic assays to explore the effects of silencing CASP3 on cell phenotypes. The descriptions of the in vitro experimental processes are provided in Supplementary material 1.

### Statistical analysis

We adopted the log-rank test to compare the overall survival curves for the two groups.

Univariate and multivariate Cox regression analysis was conducted to assess the correlation of the risk score with prognosis in low-grade glioma, and hazard ratios (HRs) and confidence intervals (CIs) were calculated. We performed all statistical analyses using R software (version 4.0). A P value < 0.05 was considered significant unless specifically defined otherwise.

## Results

### Identification of molecular subclasses of low-grade glioma

**Figure 1:**
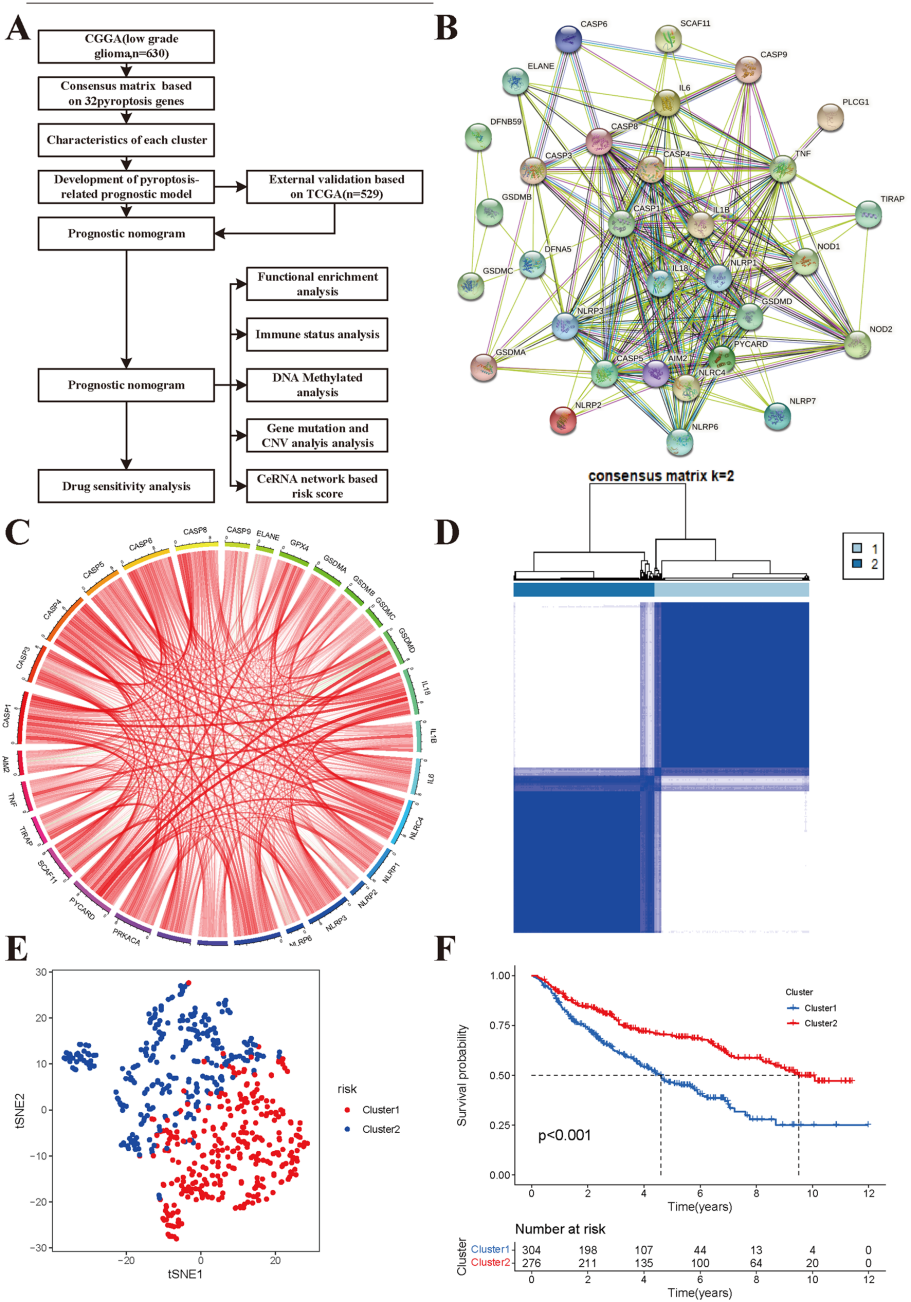
Consensus clustering identifies the molecular subtype of low-grade glioma using CGGA dataset. (**A**) Flow diagram of integrated analysis in the study. (**B**) Protein–protein interaction network of identified pyroptosis in the STRING. (**C**) The positive (red) and negative (green) correlations of pyroptosis genes using Spearman method. (**D**) Consensus clustering identified optimal number of molecular subtypes for low-grade glioma (k = 2). (**E**) The tSEN2 analysis revealed the marked two subclasses in low-grade glioma. (**F**) Kaplan–Meier survival curves of two subclasses (blue: cluster 1; red: cluster 2).

The flow diagram of the integrated analysis in the study is shown in Fig. 1A. We obtained 630 samples with mRNA expression data from the CGGA dataset, and 30 pyroptosis genes were extracted (MAD > 0.5). Supplementary material 2 shows the gene symbols and Entrez IDs of the pyroptosis genes used in this study. Using the STRING database, we constructed a protein–protein interaction (PPI) network of all pyroptosis genes, and the PPI network showed that several pyroptosis genes, including CASP1, CASP3, CASP4, CASP8, NLRP1, NLRP3, and NLRC4, were connected to more nodes than were other genes (Fig. 1B).

The expression correlations among these pyroptosis genes are presented in Fig. 1C (red: positive correlations; dark colour: negative correlations). The consensus clustering analysis indicated that all samples of low-grade glioma could be divided into two subclasses. The consensus matrix exhibited a relatively sharp and clear boundary, which indicated stable and robust clustering (Fig. 1D). We further found a two-dimensional t-sensitivity distribution that supported the subtype clustering (Fig. 1E). The specific subclass information of each sample is provided in Supplementary material 2: Table S2. Finally, we compared the overall survival curves for the two subclasses using Kaplan–Meier analysis. Our results indicated that the median overall survival time was significantly longer in Cluster 2 than in Cluster 1. Kaplan–Meier analysis indicated that the median survival time was significantly shorter in Cluster 2 than in Cluster 1 (MST: 9.62 vs. 4.35 years, P < 0.001; Fig. 1F). This result showed that the two subclasses had markedly different prognostic patterns.

### Correlations of the molecular subclasses with pyroptosis genes

**Figure 2:**
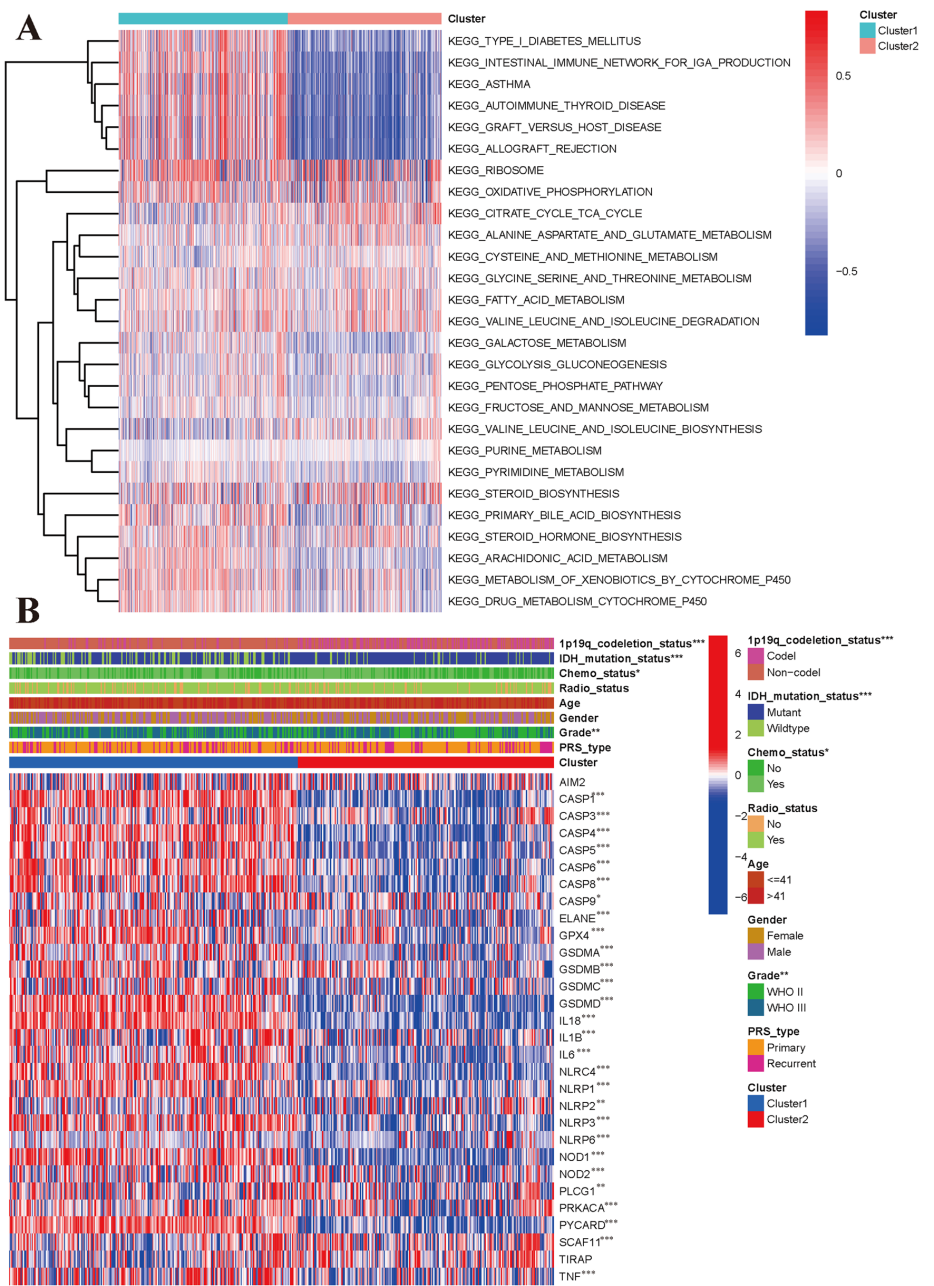
Different pathway enrichment and clinical relevance of two subclasses based on CGGA dataset. (**A**) Gene set variation analysis of two subclasses. (**B**) Expression levels of pyroptosis genes between two subclasses and its correlations with clinical parameters.

To explore the signalling pathway enrichment in the two subclasses, we performed gene set variation analysis (GSVA) by transforming the expression data from a gene-by-sample matrix to a gene set by subclass matrix for both subclasses. The results indicated that the two subclasses had different pathway enrichment profiles. Unlike Cluster 2, Cluster 1 had 78 kinds of significantly different signalling pathways (Supplementary material 2: Table S3). The upregulated pathways mainly included type 1 diabetes mellitus, intestinal immune network for IGA production, asthma, autoimmune thyroid disease, graft versus host disease, allograft rejection, and ribosome. In addition, the DNA replication, mismatch repair, ECM receptor interaction, cell cycle, and p53 signalling pathways, and some metabolic pathways were significantly enriched (Fig. 2A).

### Clinical characteristics and pyroptosis gene expression patterns of the molecular subclasses

We investigated the correlations of the two subclasses with clinical parameters (Fig. 2B). Compared with patients in Cluster 1, who had poor prognoses, patients from Cluster 2 tended to have a lower WHO stage (P < 0.001), lower rate of chemotherapy (P < 0.05), 1p19q non-codeletion status (P < 0.001), and IDH mutation status (P < 0.001). No significant differences were observed in PRS type, sex, age, or radiotherapy status (P > 0.05). Regarding the expression levels of pyroptosis genes, significant differential expression was observed between the two subclasses for all genes except AIM2 and TIRAP. All of these differentially expressed genes were upregulated in Cluster 2 (Fig. 2B). We also compared the differences in pyroptosis gene expression by WHO stage, IDH mutation status, 1p19q status, and chemotherapy history. Compared with the WHO II group, the WHO III group had sixteen upregulated genes (CASP1, CASP3, CASP4, CASP5, CASP6, CASP8, GSDMB, GSDMD, IL18, IL6, NLRC4, NOD1, NOD2, PLCG1, PRKACA, PYCARD) (Supplementary material 3: Fig. S1A). For IDH mutation status, 21 differentially expressed genes (DEGs) were found (Supplementary material 3: Fig. S1B). A total of 27 pyroptosis DEGs were found for 1p191 status (Supplementary material 3: Fig. S1C). Thirteen DEGs were found for chemotherapy history (Supplementary material 3: Fig. S1D).

We further performed differential expression analysis between Cluster 1 and Cluster 2. A total of 1053 DEGs were found; 67 genes were upregulated and 986 were downregulated in Cluster 2 (Supplementary material 2: Table S4). We performed GO and KEGG enrichment analyses using these DEGs (Supplementary material 2: Table S5 and Table S6). A total of 1033 differential functional terms were enriched, namely, 856 biological process, 116 cellular component and 61 molecular function terms. The top 30 enriched terms are presented in Supplementary material 3: Fig. S2. Most of these functions were associated with immunity. In addition, we identified 56 significant pathways by KEGG analysis (Supplementary material 3: Fig. S3), and the top five were phagosome, *Staphylococcus aureus* infection, coronavirus disease, antigen processing and presentation, and tuberculosis.

### Correlation of glioma subclass with immune status

To explore tumour heterogeneity between the two subclasses, we compared immune cell and immune function differences. Compared with Cluster 2, Cluster 1 had higher aDC, B cell, CD8+ T cell, DC, iDC, macrophage, mast cell, neutrophil, NK cell, pDC, T helper cell, Tfh cell, Th2 cell, Tfh cell, TIL, and Treg cell levels (all P < 0.001, Supplementary material 3: Fig. S4A). Similarly, Cluster 1 had higher immune function scores, including APC coinhibition, APC costimulation, CCR, checkpoint, cytolytic activity, HLA, inflammation promotion, MHC class I, parainflammation, T cell coinhibition, type I IFN response and type II IFN response scores, than Cluster 2 (all P < 0.001, Supplementary material 3: Fig. S4B).

### Establishment of the pyroptosis-related prognostic model

Initially, we performed univariate Cox regression analysis to identify prognosis-related pyroptosis genes in the CGGA cohort (Supplementary material 3: Fig. S5A). In total, 21 pyroptosis genes were found to be associated with the overall survival of low-grade glioma patients. Kaplan–Meier analysis indicated that high expression of all 21 genes (CASP1, CASP3, CASP4, CASP5, CASP6, CASP8, GSDMA, GSDMB, GSDMC, GSDMD, IL18, IL1B, IL6, NLRC4, NLRP3, NOD1, NOD2, PLCG1, PRKACA, PYCARD, SCAF11) was associated with poor OS in low-grade glioma. Furthermore, using LASSO regression in the CGGA training cohort, we identified 10 pyroptosis genes (CASP5, CASP6, CASP8, GSDMC, IL1B, IL6, NLRP3, NOD2, PLCG1, SCAF11) to establish the prognostic model (Supplementary material 3: Fig. S5B,C). We calculated the risk score for each sample using the regression coefficients of the 10 genes (Supplementary material 2: Table S7). Patients with risk scores higher than the median value were classified into the high-risk group, and the other patients were classified into the low-risk group. Compared with the patients in the low-risk group, the patients in the high-risk group tended to have advanced WHO stage (P < 0.001), recurrence (P < 0.001), a history of chemotherapy (P < 0.001), IDH mutation status (P < 0.001), and 1p19q codeletion status (P < 0.001) (Supplementary material 3: Fig. S5D). Finally, we found that glioma patients in Cluster 1, with WHO III disease, with IDH wild-type status, with 1p19q non-codeletion status, with recurrence or with a history of chemotherapy had higher risk scores (all P < 0.01, Supplementary material 3: Fig. S6).

**Figure 3:**
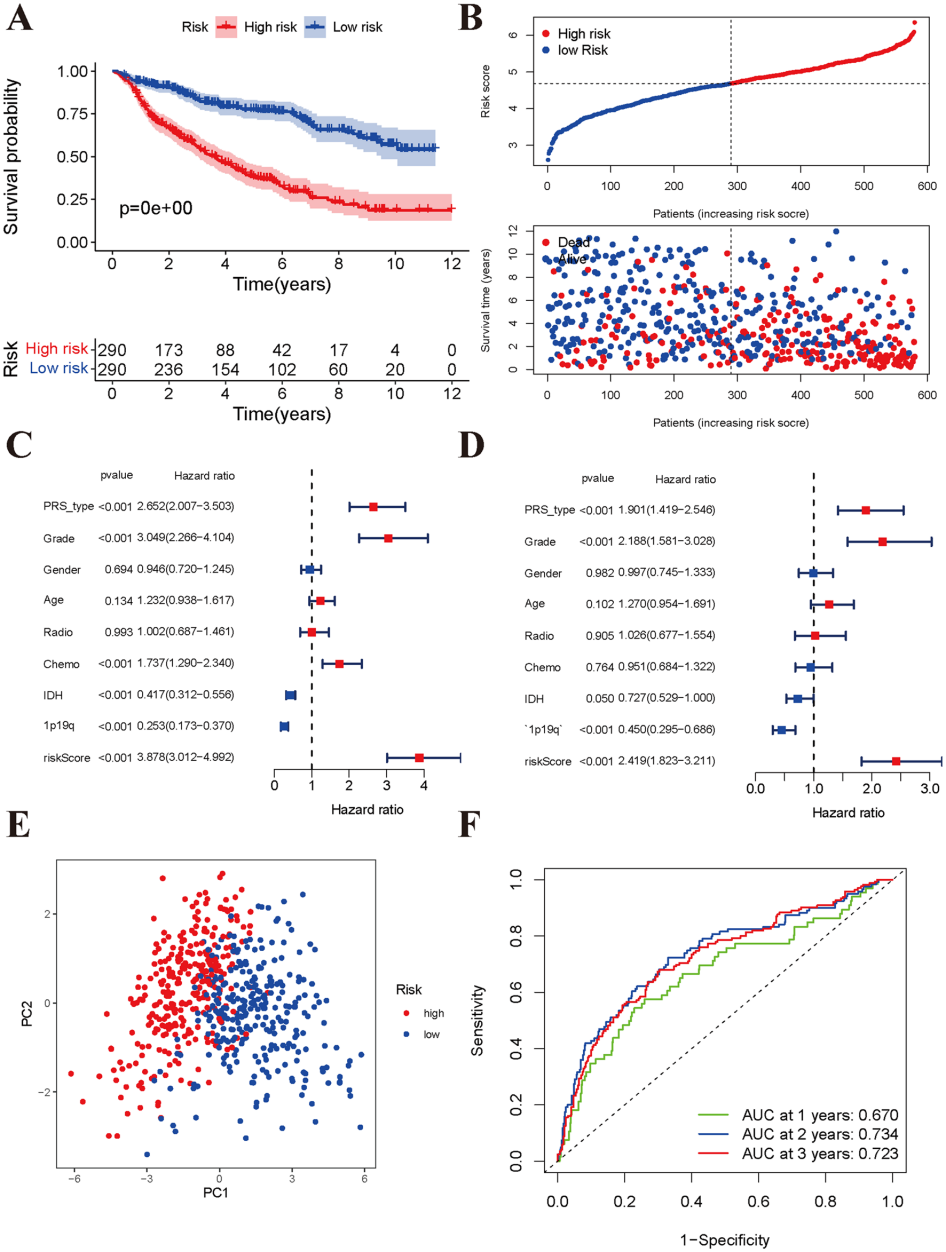
Developing a pyroptosis genes signature can predict overall survival in CGGA cohort. (**A**) Kaplan–Meier survival curves of high- and low-risk groups divided by risk score. (**B**) Distributions of risk score and survival time in different risk groups. (**C**) Univariate cox analysis identified the correlation of risk score and overall survival in low-grade glioma. (**D**) Multivariate cox analysis identified the correlation of risk score and overall survival in low-grade glioma. (**E**) Principal component analysis showed two markedly distributions for high- and low-risk groups. (**F**) The predict ability of risk score for 1-year, 2-year, and 3-year overall survival.

Kaplan–Meier analysis indicated that the high-risk group had a significantly worse OS than the low-risk group (Fig. 3A,B). Univariate Cox regression analysis indicated that the risk score was positively associated with poor OS (HR = 3. 878, 95% CI: 3.012–4.992, P < 0.001; Fig. 3C). Multivariate Cox regression analysis showed that an elevated risk score was an independent predictor of unfavourable prognosis in low-grade glioma (HR = 2.419, 95% CI: 1.823–3.211, P < 0.001; Fig. 3D). In addition, PRS type (recurrence: HR = 1.901, 95% CI: 1.419–2.546, P < 0.001) and WHO III grade (HR = 2.188, 95% CI: 1.581–3.028, P < 0.001) were positively associated with poor OS, and 1p19q non-co-deletion status (HR = 0.450, 95% CI: 0.295–0.686, P < 0.001) was negatively associated with OS. PCA also indicated that the high- and low-risk groups showed two markedly different distributions (Fig. 3E). Time-dependent receiver operating characteristic analysis was performed to evaluate the predictive ability of the prognostic model. Our results showed that the AUCs at 1 year, 2 years, and 3 years were 0.670, 0.734 and 0.723 (Fig. 3F), respectively. We further compared the OS status among the WHO stage, sex, age, IDH status, 1p19q codeletion status, radiotherapy history, and chemotherapy history subgroups. All results indicated that the OS in the high-risk group was still poorer than that in the low-risk group (Supplementary material 3: Fig. S7, all P < 0.001).

### Validation of the prognostic model

**Figure 4:**
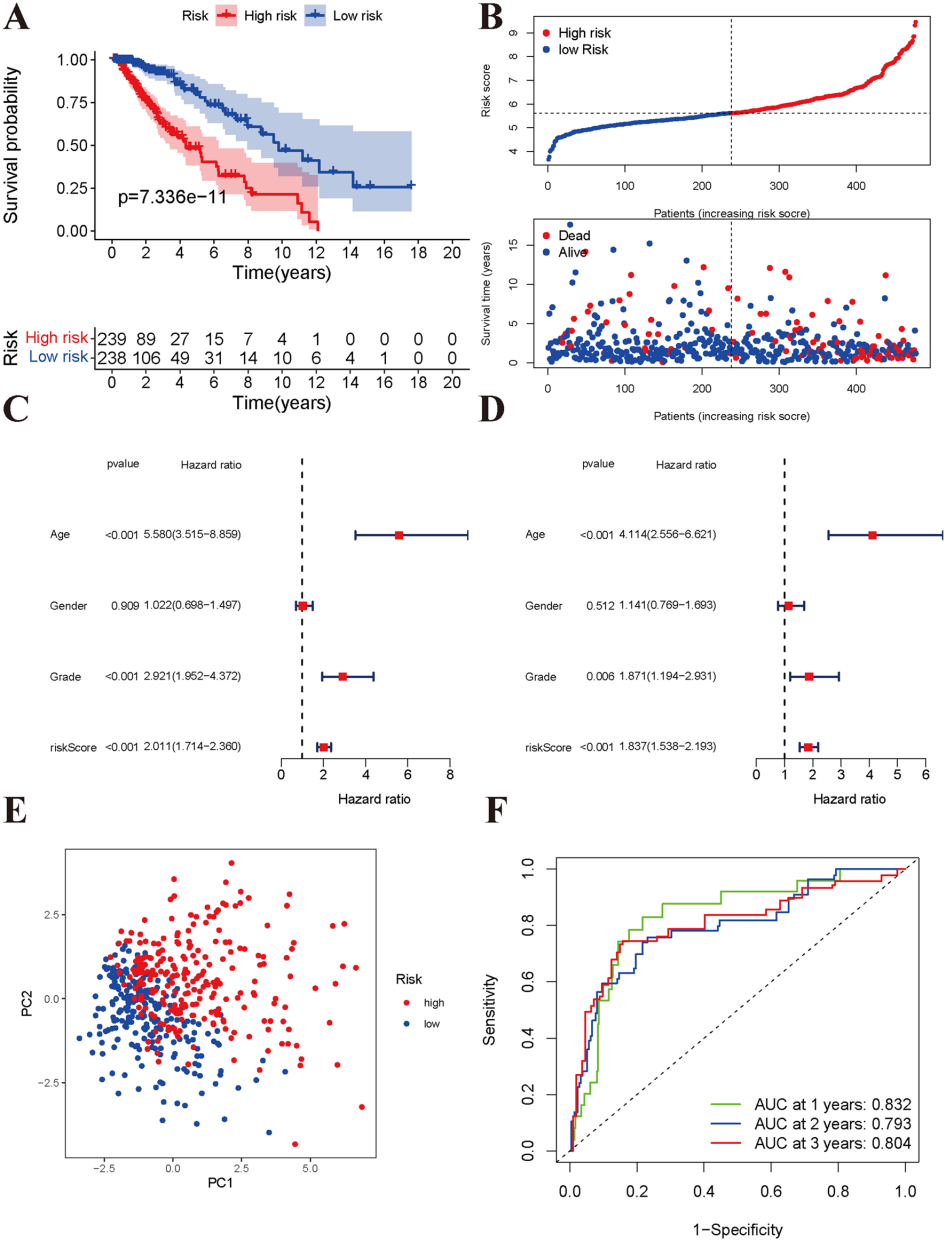
Validation of prognostic model based pyroptosis genes in TCGA cohort. (**A**) Kaplan–Meier survival curves of high- and low-risk groups divided by risk score. (**B**) Distributions of risk score and survival time in different risk groups. (**C**) Univariate cox analysis identified the correlation of risk score and overall survival in low-grade glioma. (**D**) Multivariate cox analysis identified the correlation of risk score and overall survival in low-grade glioma. (**E**) Principal component analysis showed two markedly distributions for high- and low-risk groups. (**F**) The predict ability of risk score for 1-year, 2-year, and 3-year overall survival.

To further validate the pyroptosis gene model, we also calculated the risk scores of low-grade glioma patients in the TCGA cohort. Kaplan–Meier analysis indicated a significant correlation with poor OS in the high-risk group (Fig. 4A,B). Univariate Cox regression analysis showed that an increased risk score was significantly associated with poor OS in the TCGA cohort (HR = 2.011, 95% CI: 1.714–2.360, P < 0.001; Fig. 4C). An elevated risk score was also an independent prognostic indicator in multivariate Cox regression analysis (HR = 1.837, 95% CI: 1.538–2.193, *P* < 0.001; Fig. 4D). PCA also validated the high- and low-risk distribution patterns of all patients (Fig. 4E). Furthermore, the AUCs of the risk score were 0.832 at 1 year, 0.793 at 2 years, and 0.804 at 3 years (Fig. 4F).

### Clinical application of the prognostic model

**Figure 5:**
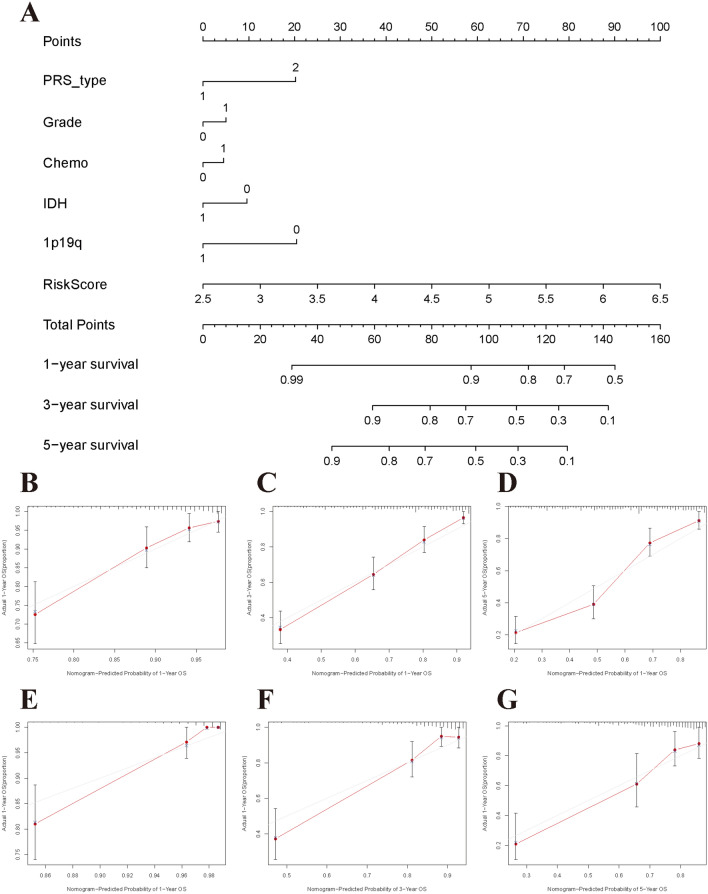
Clinical application and assessment of nomogram model based on pyroptosis genes signature. (**A**) Nomogram plot using CGGA dataset. (**B**–**D**) The 1-year, 3-year, and 5-year calibration curves in the CGGA cohort. (**E**–**G**) The 1-year, 3-year, and 5-year calibration curves in the TCGA cohort.

To further evaluate the clinical predictive value of the prognostic model, we developed a nomogram based on multivariate Cox regression analysis that included significant clinical parameters in the CGGA dataset (Fig. 5A). The calibration curves indicated that the clinical nomogram could precisely predict the 1-year, 3-year and 5-year OS of glioma patients (C-index = 0.799, Fig. 5B–D). The predictive accuracy of this nomogram was well validated in the TCGA dataset (C-index = 0.841, Fig. 5E–G).

### Functional enrichment and immune infiltration analyses

**Figure 6:**
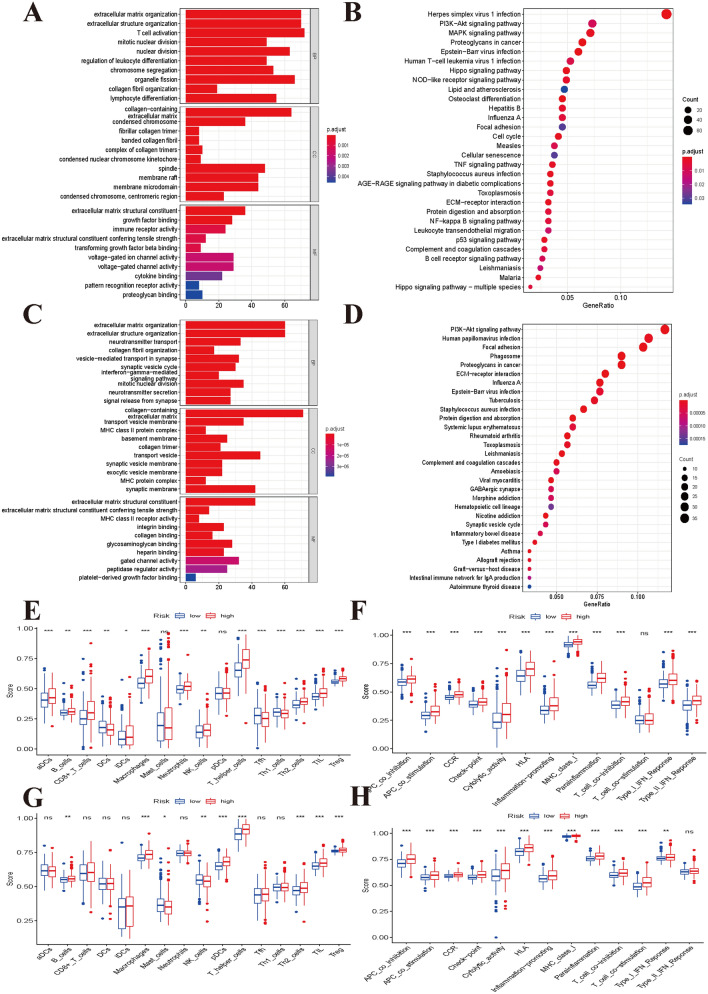
Function enrichment and immune status analyses between high- and low-risk groups (Permission for KEGG has been obtained from Kanehisa laboratories). (**A**) GO enrichment and (**B**) KEGG pathway analyses based on differentially expressed genes between high- and low- risk groups in CGGA cohort. (**C**) GO enrichment and (**D**) KEGG pathway analyses based on differentially expressed genes between high- and low- risk groups in TCGA cohort. (E) Comparisons of immune cells and (F) immune-related pathways between high- and low-risk groups in CGGA cohort. (G) Comparisons of immune cells and (H) immune-related pathways between high- and low-risk groups in TCGA cohort.

To explore the potential biological functions that affect the overall survival of low-grade glioma patients, we performed GO term and KEGG pathway enrichment analyses. We first identified DEGs between the high- and low-risk groups and then annotated the functions of the DEGs in terms of biological processes, cellular components, and molecular functions. We found 1571 DEGs in the CGGA cohort (Supplementary material 2: Table S8) and 609 DEGs in the TCGA cohort (Supplementary material 2: Table S9). The GO term and KEGG pathway enrichment analyses indicated that the CGGA and TCGA cohorts shared some enriched terms and pathways, such as extracellular matrix organization, extracellular structure organization, immune response, ECM-receptor interaction, cell adhesion molecules, PI3K-Akt signalling pathway, and Epstein–Barr virus infection (Fig. 6A–D).

We further compared the differences in immune cells and immune functions between the high- and low-risk groups in the CGGA cohort (Fig. 6E,G) and TCGA cohort (Fig. 6F,H). As shown in the box plots, the immune cell score showed a similar trend in the CGGA and TCGA cohorts. Most immune cell scores showed a tendency to be increased in the high-risk group. The differences in immune function between the different risk groups were similar in the two datasets (all P < 0.001). All immune function scores were significantly increased in the high-risk group. The 10 pyroptosis genes included in the prognostic model showed significant differences among the four immune subtypes (C3, C4, C5, C6) (Supplementary material 3: Fig. S8). We finally investigated the correlations of the signature genes with cancer stem cell-like properties (RNAss and DNAss) and the TME (stromal score, immune score, and ESTIMATE score). We found that all ten genes were negatively associated with the RNAss and the expression of CASP5, CASP6, CASP8, GSDMC, IL18, IL6m, NLRP3, and NOD2 and that all genes except PLCG1 and SCAF11 showed positive associations with the DNAss, stromal score, immune score, and ESTIMATE score. PLCGa and SCAF11 were negatively associated with the stromal score, immune score, and ESTIMATE score. SCAF11 was the only gene that was negatively associated with the DNAss (Supplementary material 3: Fig. S9).

### Profiling of pyroptosis gene alterations

**Figure 7:**
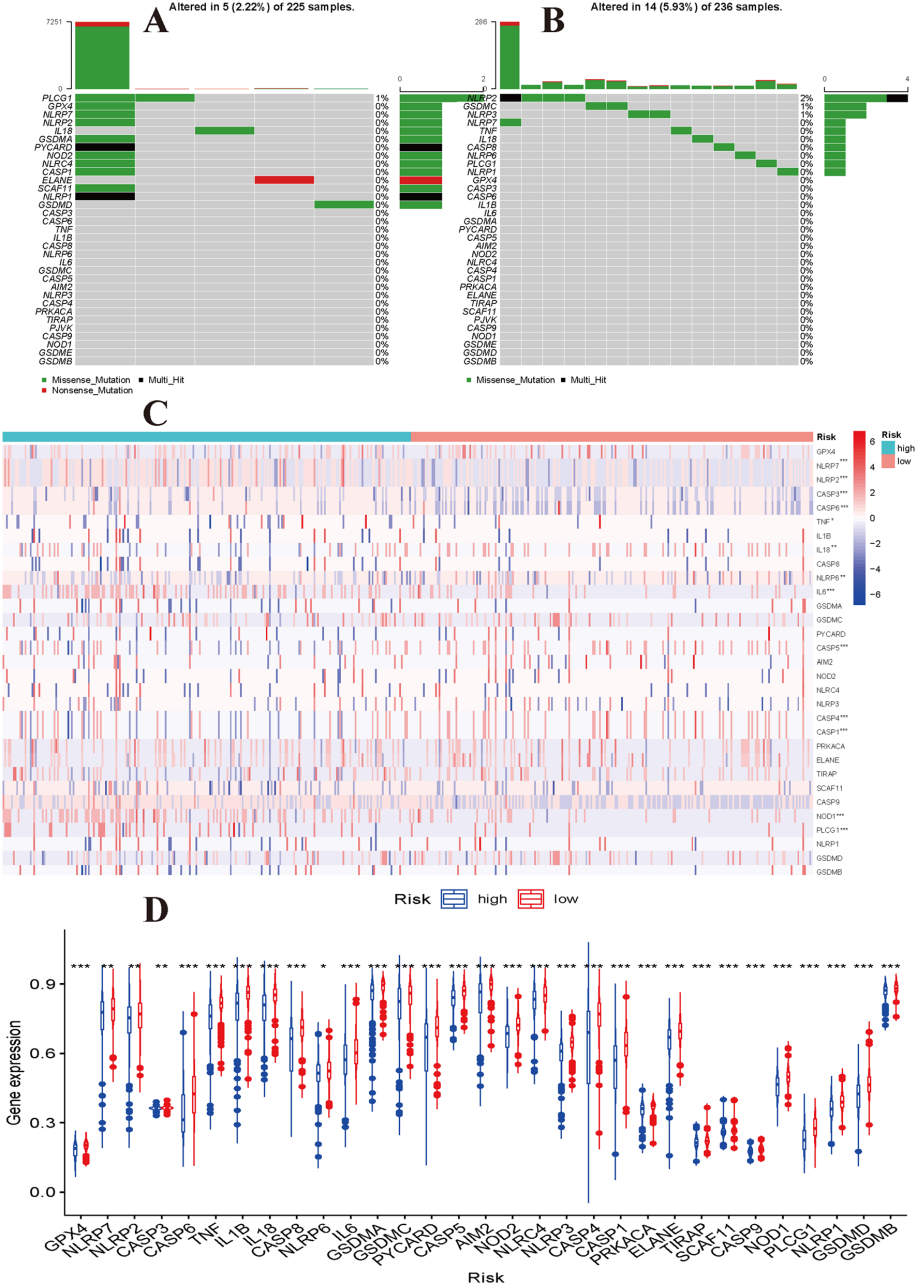
Molecular alterations of pyroptosis genes in TCGA dataset. (**A**) The mutations frequencies in low-risk group and (**B**) in high-risk group. (**C**) Somatic copy number differences between high- and low-risk groups. (**D**) The differential expression levels of DNA methylation between high- and low-risk groups.

Molecular alterations in pyroptosis-related genes were also evaluated based on the high- and low-risk groups in the TCGA dataset. PLCG1 was the only gene with alteration in the low-risk group, and NLRP2, GSDMC and NLRP3 were the genes with alterations in the high-risk group. All altered genes had an alteration frequency of less than or equal to 2% (Fig. 7A,B). Somatic copy number alteration analysis indicated significant differences among the pyroptosis genes. Among these genes, the copy number variation in IL18, NLRP6, IL6, CASP5, CASP4, CASP1, and NOD1 was significantly increased and that of TNF, NLRP2, NLRP7, CASP3, and CASP6 was significantly decreased in the high-risk group (Fig. 7C). In addition, differences in the DNA methylation levels of the pyroptosis genes between the high- and low-risk groups were assessed. The results showed that the overall DNA methylation levels of all pyroptosis genes were significantly higher in the high-risk group than in the low-risk group (Fig. 7D).

### Construction of a ceRNA network based on the prognostic signature

**Figure 8:**
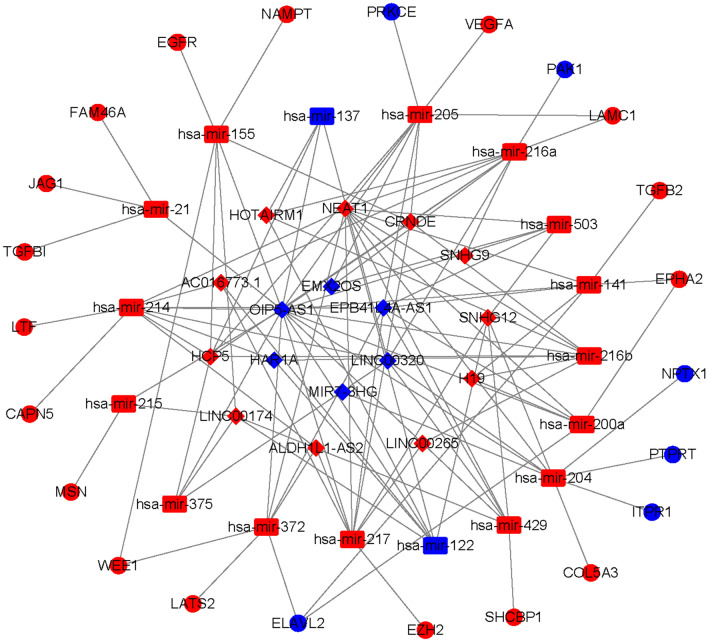
The ceRNA regulation network based on differentially expressed mRNA, lncRNA, and miRNA between high- and low-risk groups in TCGA dataset (red: up-regulation. blue: down-regulation; circle: mRNA, rhombus: lncRNA, rectangle: mirRNA).

To explore differences in the lncRNA–miRNA–mRNA regulatory network between the high- and low-risk groups, we constructed a ceRNA network based on the differentially expressed mRNAs, lncRNAs and miRNAs between the high- and low-risk groups. We identified 217 downregulated mRNAs, 493 upregulated mRNAs, 71 downregulated lncRNAs, 110 upregulated lncRNAs (Supplementary material 2: Table S10), 77 downregulated miRNAs and 94 upregulated miRNAs (Supplementary material 2: Table S11). Finally, 23 mRNAs (17 upregulated and 6 downregulated), 17 lncRNAs (11 upregulated and 6 downregulated) and 17 miRNAs (15 upregulated and 2 downregulated) were included in the ceRNA network (Fig. 8). The Kaplan–Meier curves suggested that 15 lncRNAs (positive correlation: AC016773.1, ALDH1L1-AS2, CRNDE, LINC00519, GCP5, HOTAIRM1, LINC00174, LINC00265, NEAT1, SNHG9, SNHG12; negative correlation: EPB41L4A-AS1, HAR1A, LINC00320, MIR7-3HG, OIP5-AS1; Supplementary material 3: Fig. S10), 23 mRNAs (Supplementary material 2: Table S12 and Supplementary material 3: Fig. S11) and 11 miRNAs (miR-21, miR-137, miR-141, miR-155, miR-200a, miR-204, miR-214, miR-215, miR-216a, miR-217, and miR-429; Supplementary material 3: Fig. S12) were associated with OS in glioma patients.

### Drug sensitivity analysis

To identify potential target molecular compounds, we performed drug sensitivity analysis. We identified 155 pairs of significant gene-drug correlations (Supplementary material 2: Table S13). There were 5 pairs with a correlation coefficient > 0.5 or < − 0.5.

**Figure 9:**
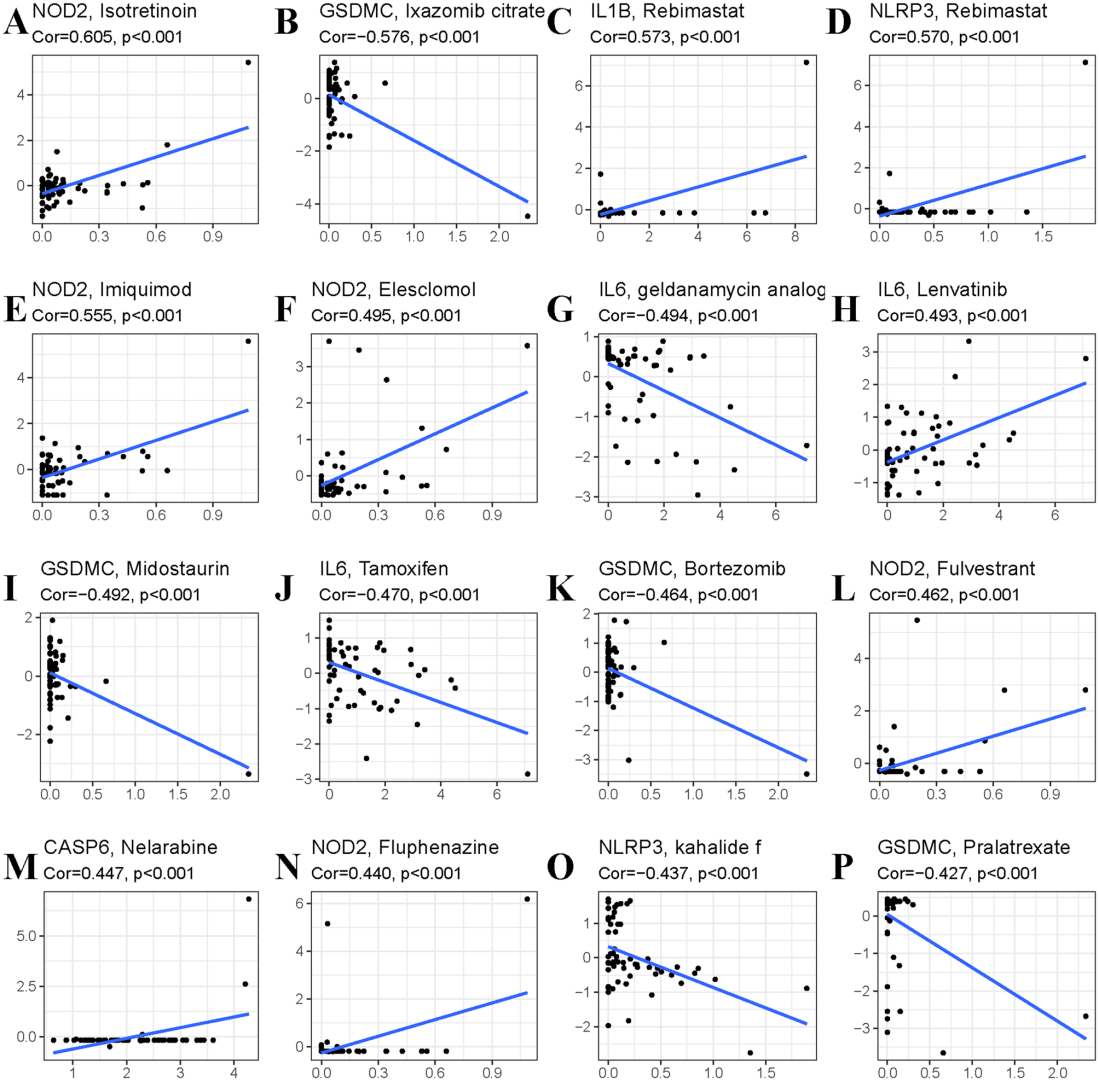
Top 16 potential compounds related with pyroptosis genes. (**A**) NOD2 and isotretinoin. (**B**) GSDMC and Ixazomib. (**C**) IL1B and Rebimastat. (**D**) NLRP3 and Rebimastat. (**E**) NOD2 and Imuiquimod. (**F**) NOD2 and Elesclomol. (**G**) IL6 and Geldanamycin analog. (**H**) IL6 and Lenvatinib. (**I**) GSDMC and Midostaurin. (**J**) IL6 and Tamoxifen. (**K**) GSDMC and Bortezomib. (**L**) NOD2 and Fulvestrant. (**M**) CASP6 and Nelarabine. (**N**) NOD2 and Fulvestrant. (**O**) NLRP3 and Kahalide. (**P**) GSDMC and Pralatrexate.

The NOD2-isotretinoin, IL1B-rebimastat, NLRP3-rebimastat, and NOD2-elesclomol pairs showed drug sensitivity. The GSDMC–ixazomib citrate pair showed drug resistance (Fig. 9).

### CASP8 silencing inhibited the malignant progression of glioma cells

**Figure 10:**
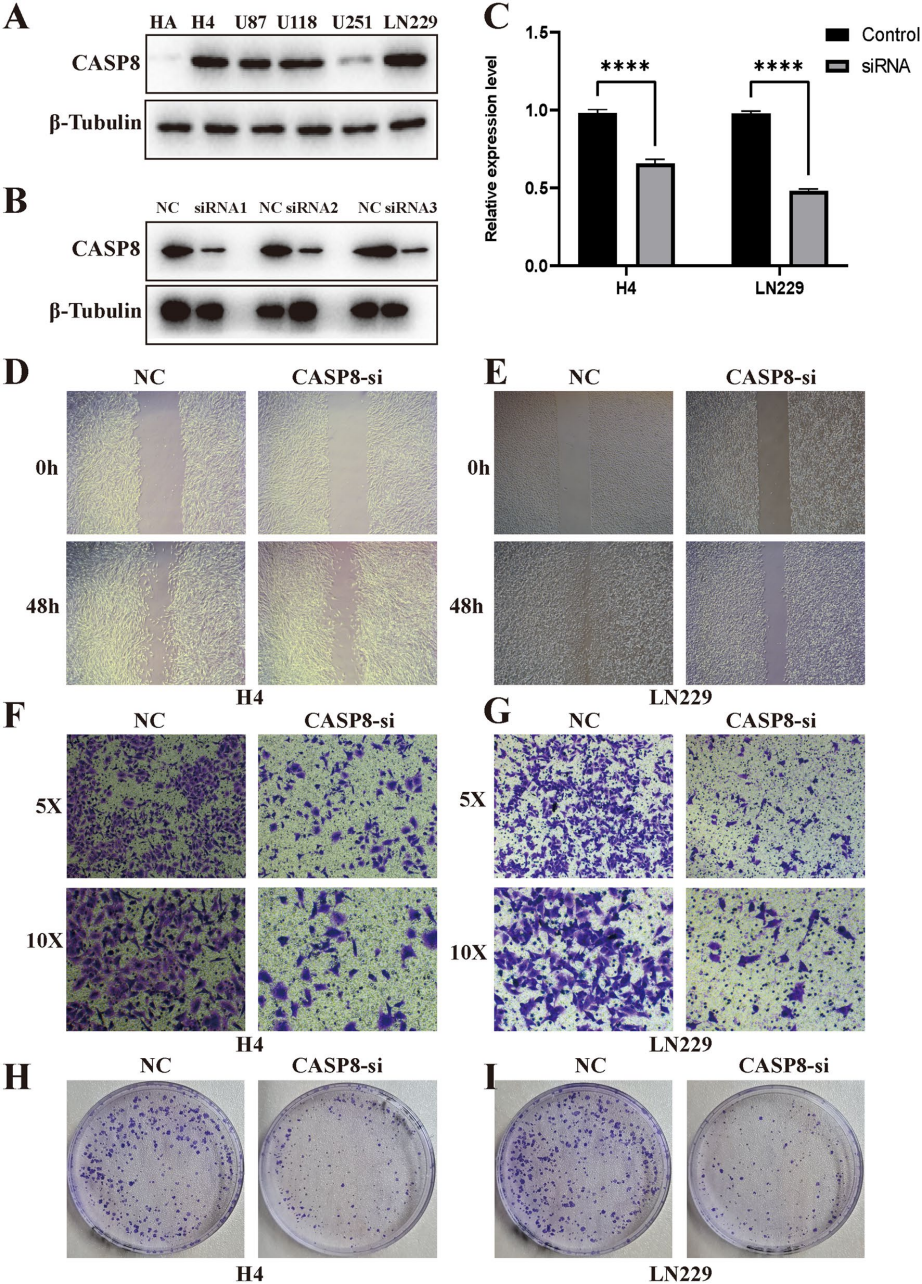
CASP8 promotes progression of glioma cells. (**A**) Expression levels of CASP8 in glioma cell lines. (**B**) The western blot of CASP2 in U87, LN229, H4 cell lines after siRNA. (**C**) The mRNA expression level of CSAP8 in H4 and LN229 after siRNA. (**D**,**E**) CASP8 silencing inhibited migration of glioma cells. (**F**,**G**) CASP8 silencing inhibited invasion of glioma cells. (**H**,**I**) CASP8 silencing inhibited growth of glioma cells.

We used the following criteria to select the validated genes: (1) genes differentially expressed between tumour and normal tissue; (2) genes included in the prognostic model; and (3) genes that were rarely reported previously (Supplementary material 3: Fig. S13). We first detected the expression of CASP8 in glioma cell lines using Western blot analysis (Fig. 10A,B) and found that CASP8 expression was highest in LN299 cells. We generated CASP8-si LN229, H4 and U87 glioma cells. qPCR analysis indicated that the mRNA expression of CASP8 was significantly downregulated in CASP8-si H4 and LN229 cells (Fig. 10C). Furthermore, silencing CASP8 inhibited the migration and invasion of glioma cells (Fig. 10D–G). The clonogenic assay showed that CASP8 silencing also inhibited cell growth (Fig. 10F–G). These results suggested that CASP8 silencing suppressed the malignant progression of glioma cells.

## Discussion

In the present study, we found that low-grade glioma can be categorized into two molecular subtypes based on the expression levels of 30 pyroptosis genes, and markedly different survival outcomes were observed between the two subclasses. The traditional classification system based on clinical parameters have been updated several times. However, the traditional classification based on histology also has some limitations. The primary limitation is mainly due to interobserver heterogeneity^2^. It was previously reported that there was only approximately 50% agreement between different neuropathologists in reviewing cases, specifically astrocytoma and oligodendroglioma cases^16^. Genetic studies and the associated findings enable us to better understand how tumours differ in clinical outcomes and molecular patterns and to facilitate the effective treatment of tumour subtypes based on gene expression features.

We also found that some immune-related pathways were enriched in the poor prognosis group. Furthermore, the poor prognosis group showed elevated immune cell levels and immune-related functions, such as immune checkpoint activity, inflammation promotion, and parainflammation. It has been reported that pyroptosis represents an antitumour immune function in cancers, which means that pyroptosis can induce inflammation, triggering robust antitumour immunity and synergizing with immune checkpoint blockade^17^. Moreover, some key pathways, such as oxidative phosphorylation, PPAR signalling pathway, primary immunodeficiency, and citrate cycle (TCA cycle), which have been reported to be involved in glioma progression, were highly enriched in the poor prognosis group^18–21^. These results suggested that pyroptosis genes can accurately differentiate low-grade glioma patients into two-dimensional distributions.

We developed a prognostic model based on 10 pyroptosis genes (CASP5, CASP6, CASP8, GSDMC, IL1B, IL6, NLRP3, NOD2, PLCG1, SCAF11). This prognostic model was well validated in an independent cohort, and the 1-year, 2-year, and 3-year AUCs were 0.670, 0.734 and 0.723, respectively. This predictive ability showed the moderate discernibility of the model. Furthermore, using the clinical parameters and risk scores obtained from the 10 genes included in the model, we developed a nomogram for estimating an individual’s overall survival probability. The results from the training dataset and validation dataset showed high consistency. Our results suggested that the established prognostic model has clinical value.

The cell death via pyroptosis involves two biological mechanisms. The classical pyroptosis mechanism involves the assembly of inflammasomes. Inflammasomes are macromolecular protein complexes that are necessary for inflammation in the cytoplasm and recognize danger signal molecules such as those released by bacteria and viruses^22^. Inflammasomes are mainly composed of pattern recognition receptors (PRRs), apoptosis-associated speck-like protein (ASC) and pro-caspase-1 precursors^23^. PRRs are receptor proteins responsible for recognizing different signal stimuli in cells. They are mainly composed of nucleotide-binding oligomerization domain-like receptor protein (NLRP) 1, NLRP3, nucleotide-binding oligomerization domain-like receptor protein C4 (NLRC4), absent in melanoma 2 (AIM2) and other components^24^. ASC is an adaptor protein that is mainly composed of an N-terminal pyrin domain (PYD) and a C-terminal caspase activation and recruitment domain (CARD)^25^. Procaspase-1 is an effector molecule that can specifically cleave GSDMD after activation. After the danger signal sensor NLR1, NLRP3 or AIM2 recognizes a danger signal molecule, the N-terminal PYD interacts with the N-terminal PYD of the adaptor protein. ASC then recruits Caspase-1 through interaction with the CARD domain to complete the assembly of the inflammasome^26^. This method of cell death mediated by Caspase-1 is called the classical pathway of pyroptosis^27^. The nonclassical pyroptosis pathway is mainly mediated by Caspases-4, -5, and -11. After cells are stimulated by bacterial LPS, Caspases-4, -5, and -11 directly bind to bacterial LPS and are activated^28^. Activated Caspases-4, -5, and -11 specifically cleave GSDMD and alleviate the intramolecular inhibition^29^. The interaction of the GSDMD-N-terminus with cell membrane phospholipids causes pore formation in the cell membrane, cell swelling and cell rupture and induces pyroptosis; the GSDMD-N-terminus can also activate Caspase-1 by activating the NLRP3 inflammasome^30^. Activated Caspase-1 stimulates the maturation of the IL-18 and IL-1β precursors, and IL-18 and IL-1β are secreted into the extracellular space and amplify the inflammatory response. Yang et al. found that in the nonclassical Caspase-11-dependent pathway, gap junction protein-1 (Pannexin-1) can be cleaved and that cleavage of Pannexin-1 can activate its own channel and release ATP, which induces pyroptosis^31^. Lamkanfi et al. found that in the nonclassical Caspase-11-dependent pathway, Pannexin-1 cleavage can also activate the NLRP3 inflammasome, which in turn activates Caspase-1 and induces pyroptosis^32^. In our study, we found that the prognostic model included CASP5, CASP6, and CASP8 along with IL18, NLRP3, and GSDMC.

These results indicated that two biological mechanisms are involved in the development and progression of low-grade glioma. Further research is required to illustrate the specific molecular mechanisms.

We divided low-grade glioma patients into high- and low-risk groups using the estimated risk score and investigated the differences in genomic patterns and clinical features between the two risk groups. Our results indicated that the enriched functions and pathways were markedly different between the high- and low-risk groups. However, the results in two independents datasets showed that these risk groups shared some similar functional enrichment and signalling pathways, such as extracellular matrix organization, focal adhesion, ECM-receptor interaction, and GABAergic synapse. Immune infiltration is very closely associated with the progression of tumours^33^. Our results showed that the immune cell and immune function scores were significantly elevated in the high-risk group and that the poor prognosis group also had a higher risk score than the favourable prognosis group. These results showed that the molecular subtypes and risk classifications were stable and robust. Subsequently, we explored the differences in alterations, CNVs, and DNA methylation levels of pyroptosis genes between the high- and low-risk groups. We found that these genomic patterns were significantly different between the two risk groups, providing some molecular basis for the changes corresponding to poor clinical outcomes. Finally, we constructed a ceRNA regulatory network that identified several key lncRNA–miRNA–mRNA regulatory axes. The ceRNA network identified several key lncRNA–miRNA–mRNA regulatory axes: FAM181A-AS1-miR-21-(MAP2K3, JAG1, TGFBI, and FAM24A), CRNDE-miR-155-(DPYA1L1, NAMPT, TRIP13, IKBIP, SPL1, EGFR, WEE1), NEAT1-miR-200a-(EPHA2, DPY19L1, PTPRD, LATS2, and ELAVL2). Survival analysis further suggested regulatory correlations: elevated expression of FAM18A-AS1 and miR-21 was associated with poor prognosis in low-grade glioma, and high expression of EGFR, WEE1, MAP2K3, JA1, and EPHA2 was associated with poor prognosis. Previous experiments have reported the promoting role of miR-21 in glioma^34,35^, and upregulation of EGFR and EPHA2 is associated with the development and progression of glioma^36–39^. Drug sensitivity analysis indicated that NOD2, ELANE, NLRP3, CASP3, and PRKACA showed sensitivity to small molecule drugs, and that PRKACA and IL6 showed resistance to some compounds. A previous study reported that the migration ability of glioma cells was reduced after inhibition of the NLRP3 inflammasome by beta-hydroxybutyrate^40^. These results may provide some guidelines for clinical therapy.

In conclusion, our results indicate that pyroptosis genes can be used to classify low-grade glioma patients into two subgroups. The established prognostic model based on 10 pyroptosis genes can not only predict the prognosis of low-grade glioma patients but also reflect the molecular alterations, immune status, and stem cell-like properties of the high- and low-risk groups. Classification based on the risk score of the prognostic signature revealed a lncRNA–miRNA–mRNA regulatory network. The correlations of the signature genes with drug sensitivity may provide a rationale for clinical treatment. Finally, our study uncovers the biological function and clinical relevance of pyroptosis in the occurrence and progression of low-grade glioma and may offer some risk management and treatment strategies. Future research should focus on molecular mechanisms and therapeutic targets.

## Supplementary Information

Supplementary Information 1.

Supplementary Information 2.

Supplementary Information 3.

Supplementary Information 4.
