# Supplementary material for: Integrative analysis reveals the functional implications and clinical relevance of pyroptosis in low-grade glioma
Source: Sci Rep. 2022 Mar 16;12:4527. doi: 10.1038/s41598-022-08619-w (PMC8925295; doi:10.1038/s41598-022-08619-w)
Supplement: Supplementary file 3 — Supplementary Information 3. [file 41598_2022_8619_MOESM3_ESM.docx]

**Supplementary material 3** Figure S1-S13

**
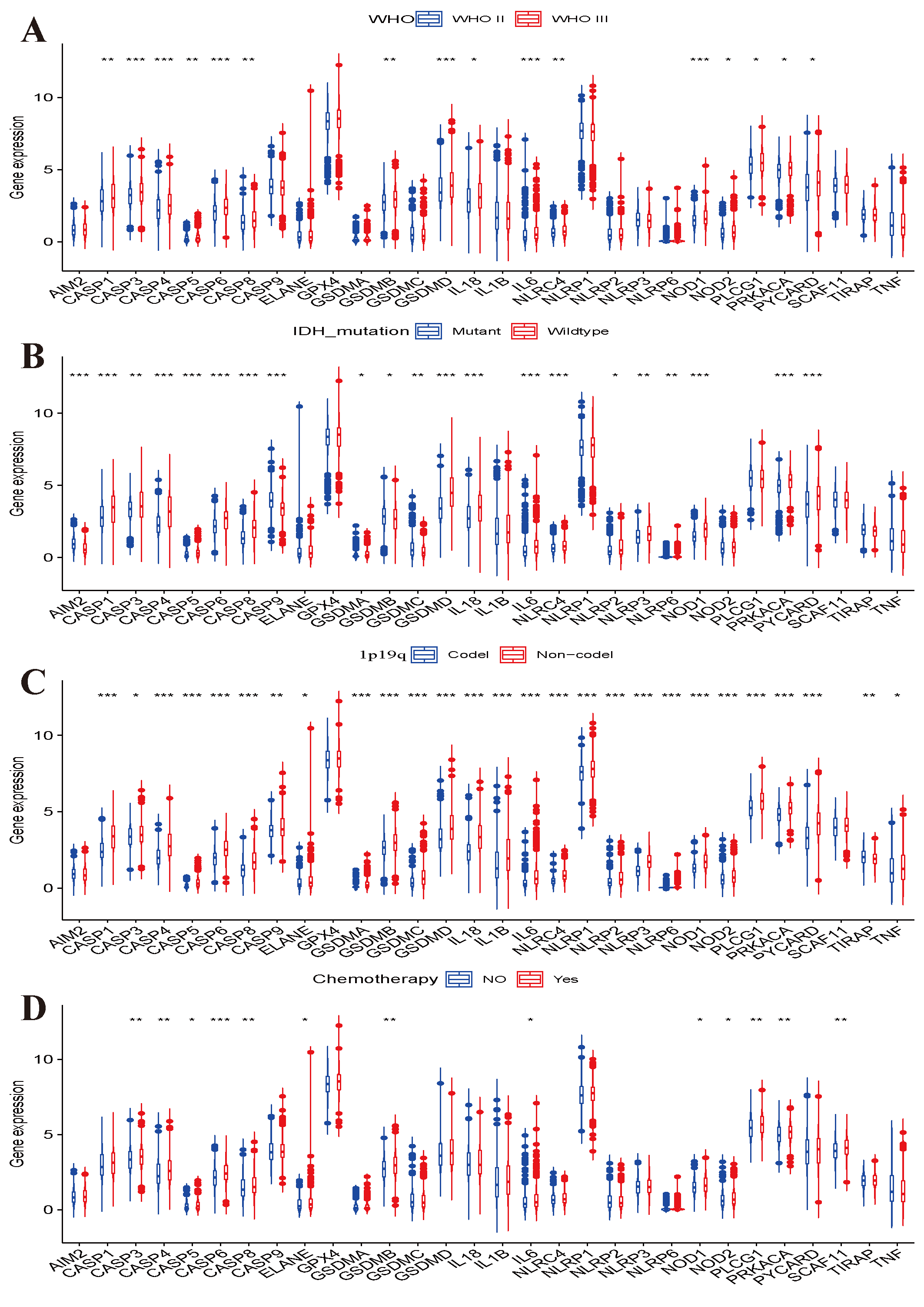
**

**Figure S1** Correlations of pyroptosis genes expression with clinical parameters. **(A)** WHO II vs WHOIII. **(B)** IDH: mutations vs wildtype. **(C)**1p19q_status: codeletion vs non-codeletion. **(D)** Chemotherapy: Yes vs NO

**
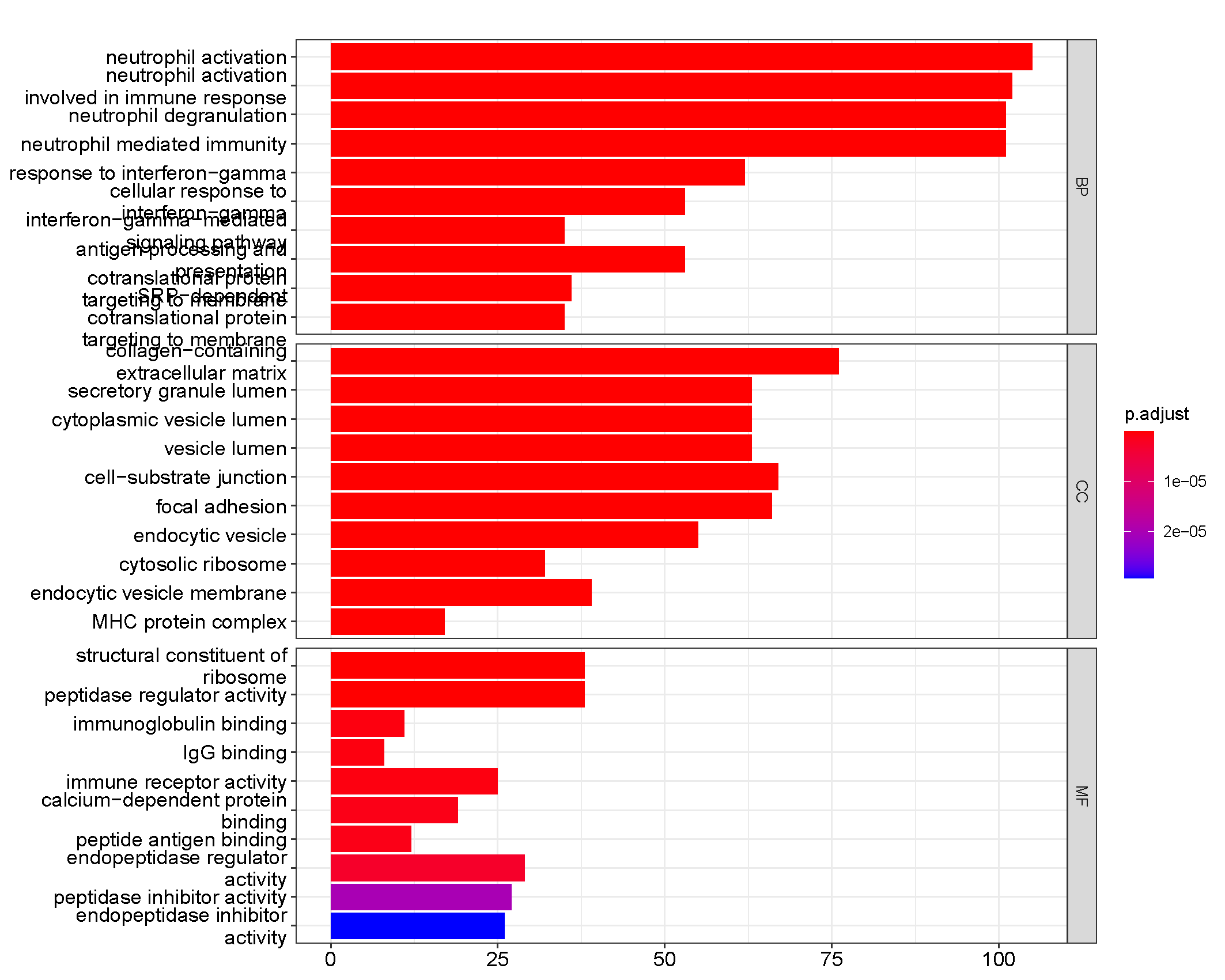
**

**Figure S2** GO enrichment analysis for differentially expressed genes based on subclasses.

**
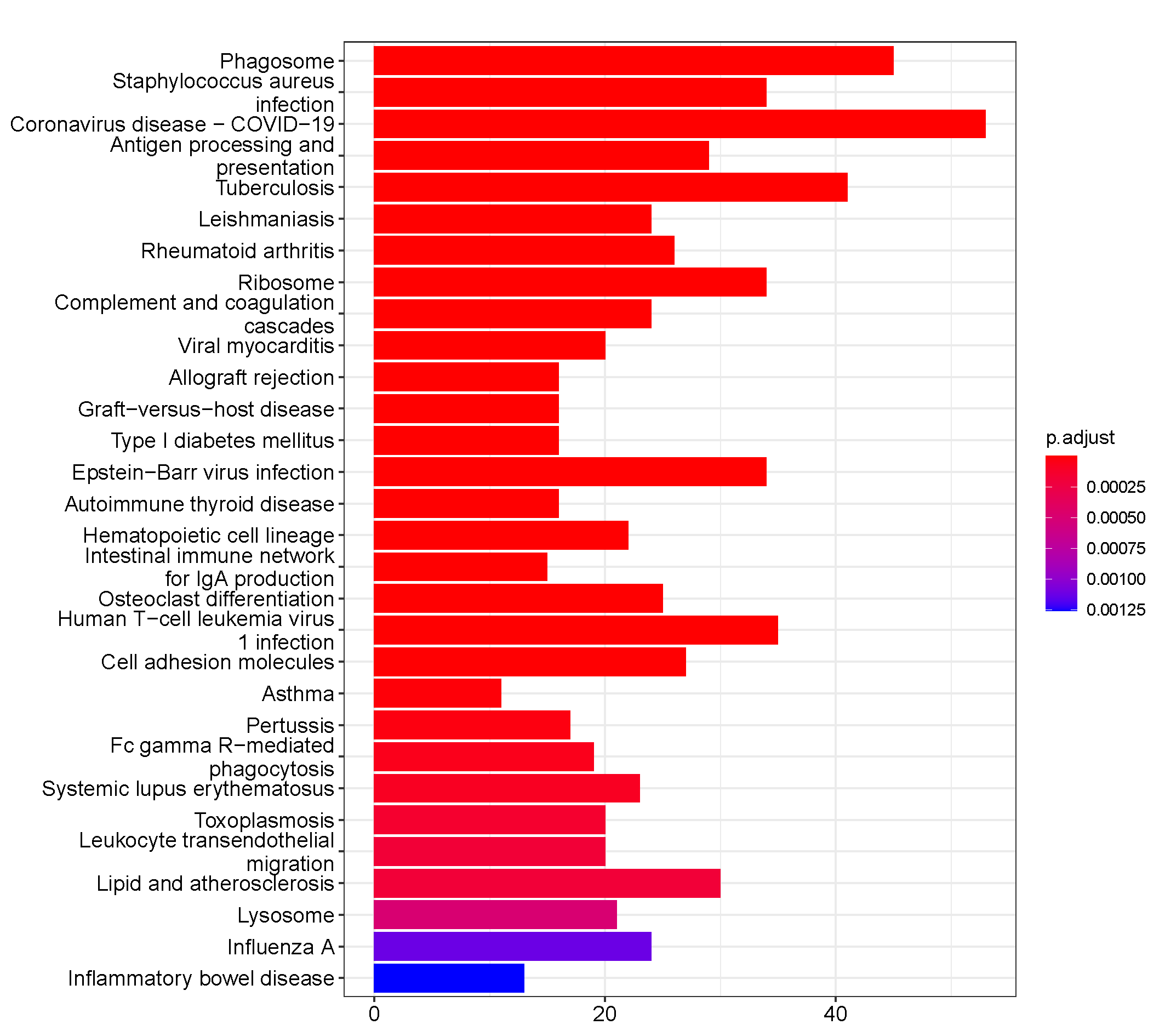
**

**Figure S3** KEEG pathways analysis for differentially expressed genes based on subclasses.

**
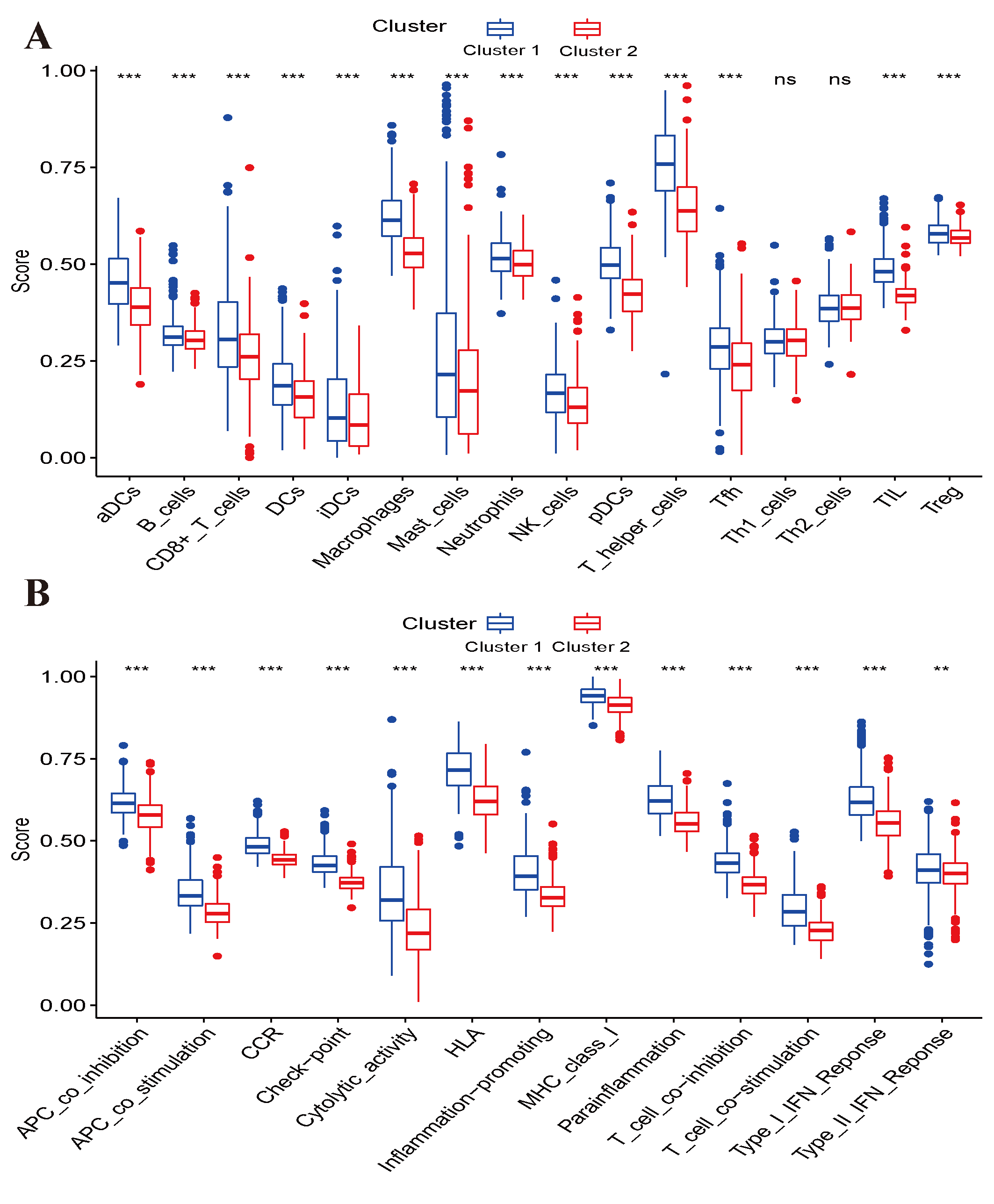
**

**Figure S4** Correlation of low-grade glioma subclasses with immune infiltration. **(A)** Immune cells. **(B)** immune function

**
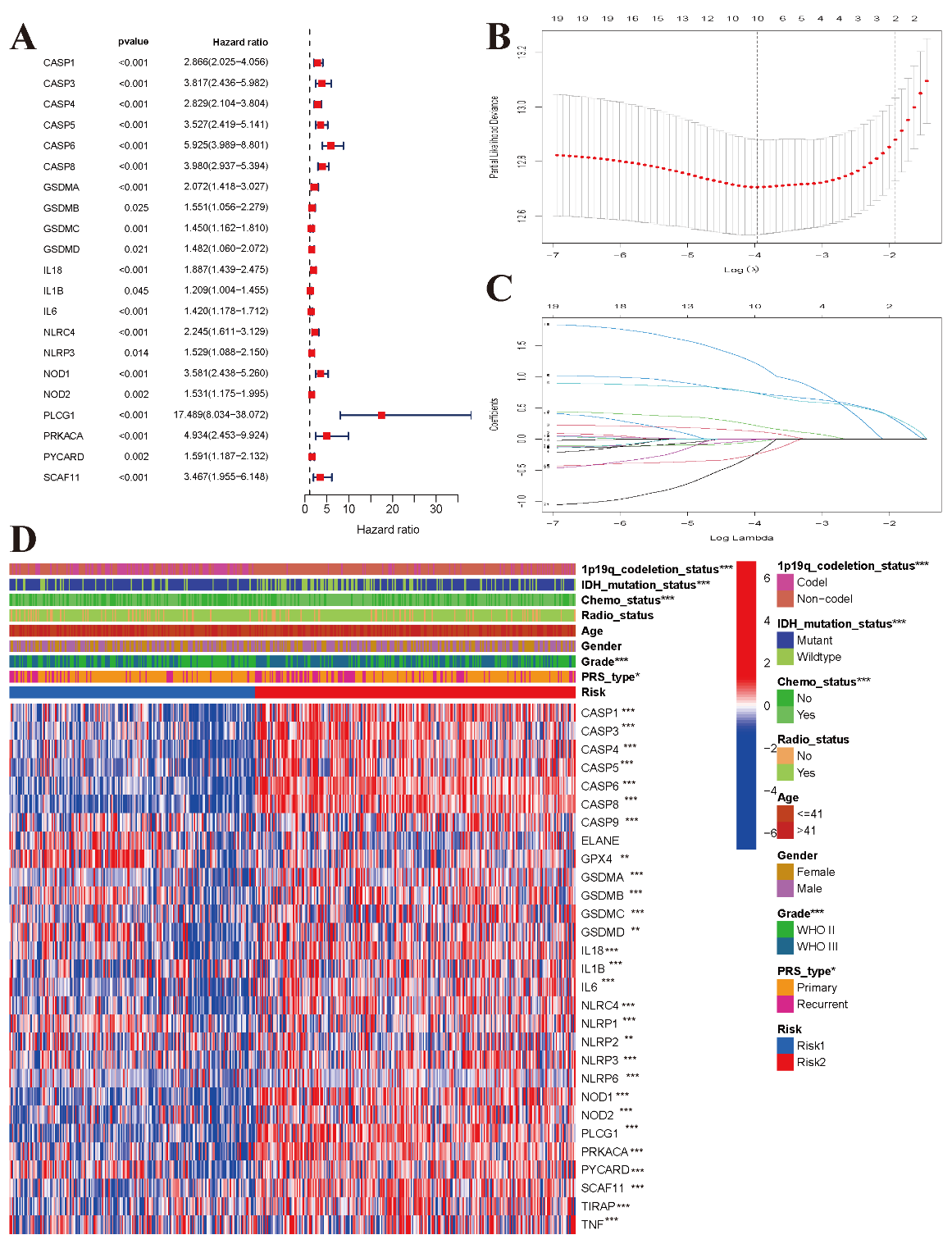
**

**Figure S5** Identification of 21 genes risk signature for OS by LASSO regression in the CGGA cohort. **(A)** univariate cox regression of OS for 30 pyroptosis-related genes. **(B)** Cross-validation for tuning parameters selection int the LASSO regression. **(C)** LASSO regression of the 10 OS-related genes. **(D)** Heatmap showed the association between risk scores and clinical parameters and differentially expressed genes of high- and low-risk group.


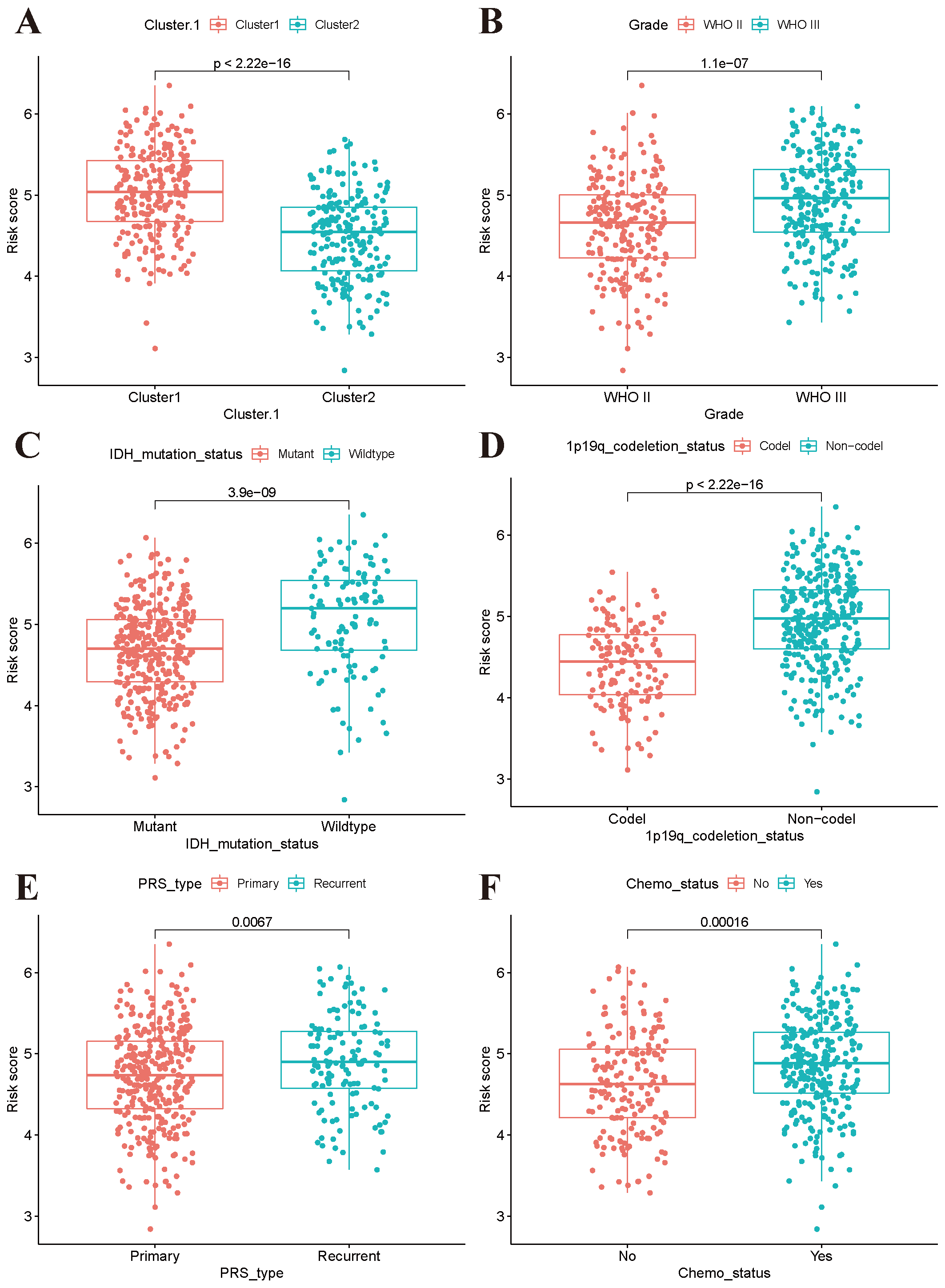


**Figure S6** Comparisons of risk score among different clinical parameters. **(A)** Subclasses: Cluster 1 vs Cluster 2. **(B)** Grade: WHO II vs WHO III. **(C)** IDH status: Mutant wildtype. **(D)** 1p19q status: Codeletion vs non-codeletion. **(E)** primary vs recurrent. **(F)** chemotherapy: No vs Yes.


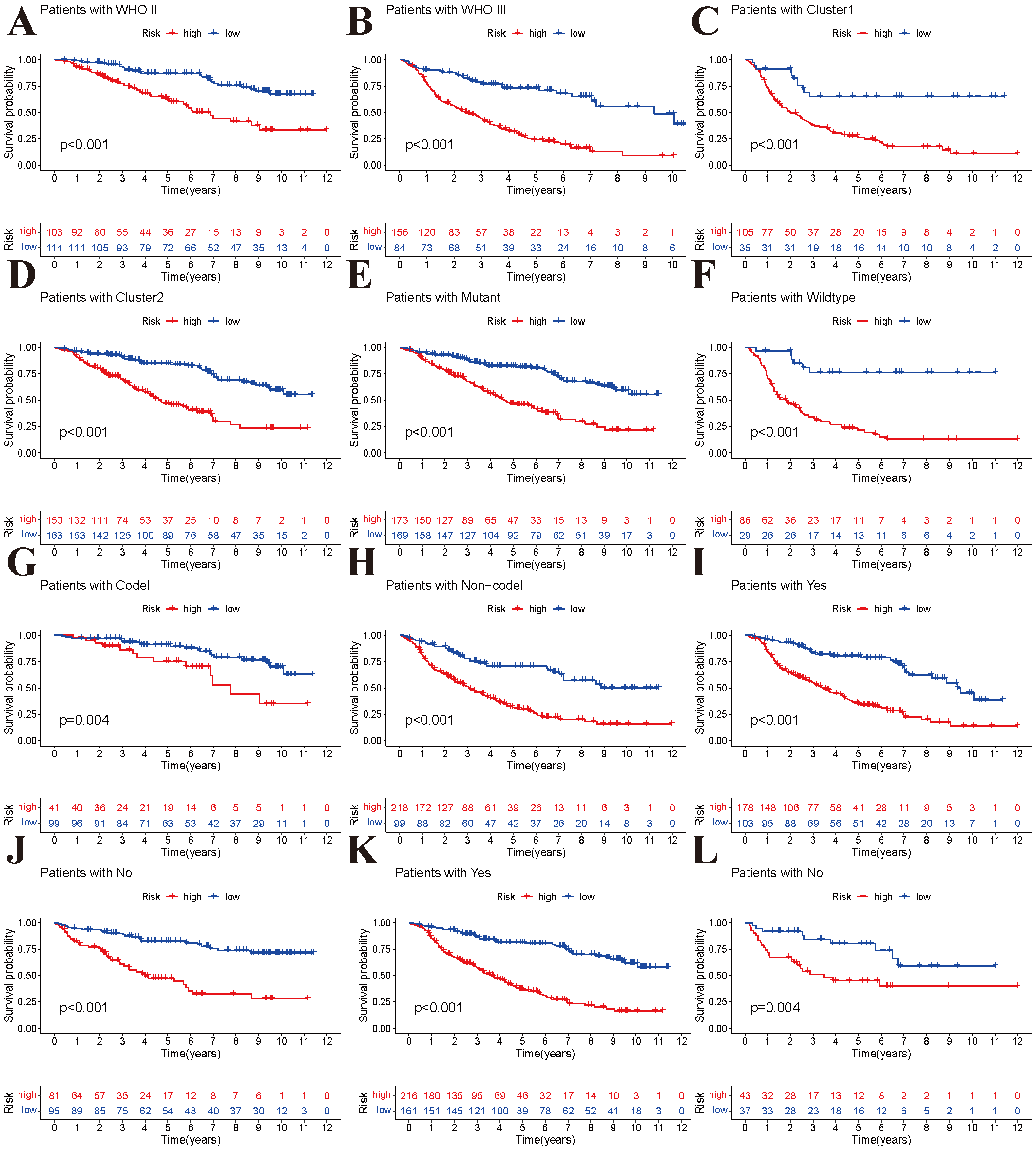


**Figure S7** Stratified analysis of OS based on risk score. **(A)** WHO II. **(B)** HWO III. **(C)** Cluster 1. **(D)** Cluster 2. **(E)** IDH mutation. **(F)** IDH wildtype. **(G)** Codeletion. **(H)** non-codeletion. **(I)** Chemotherapy: Yes. **(J)** Chemotherapy: No. **(K)** Radiotherapy: Yes. **(L)** Radiotherapy: No.

**
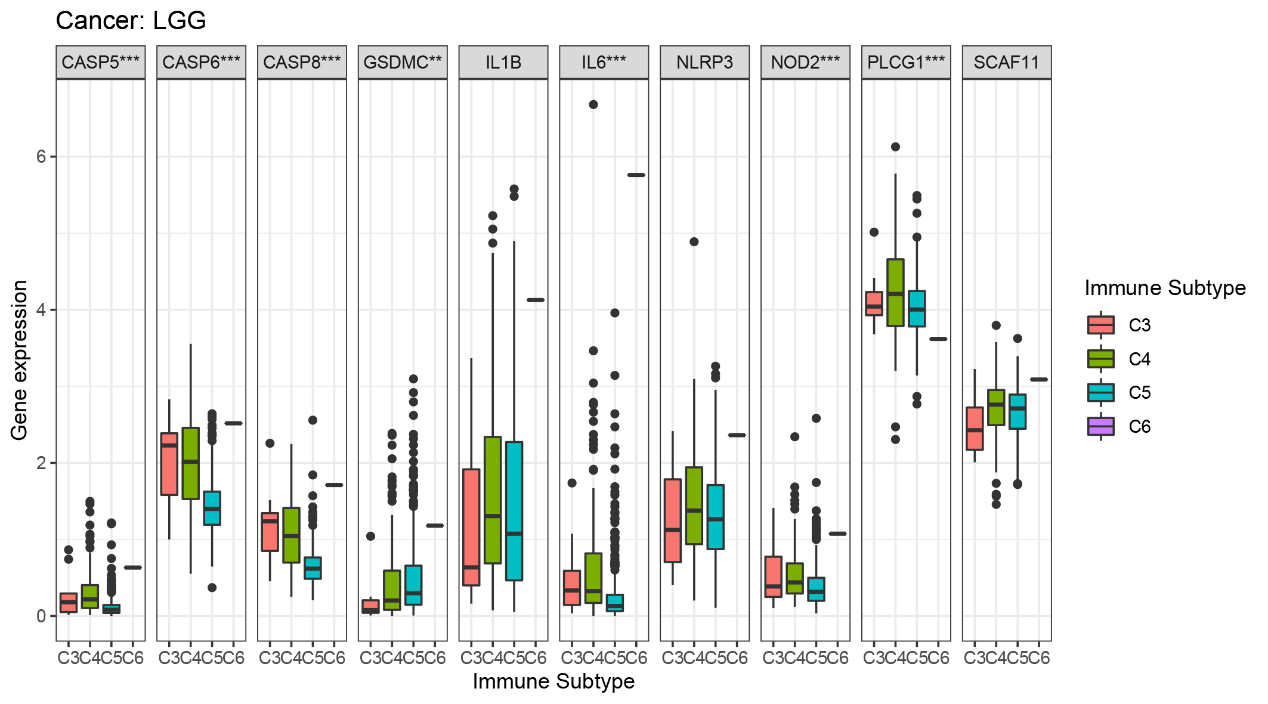
**

**Figure S8** Comparisons of 10 signature genes among different immune subtype.

**
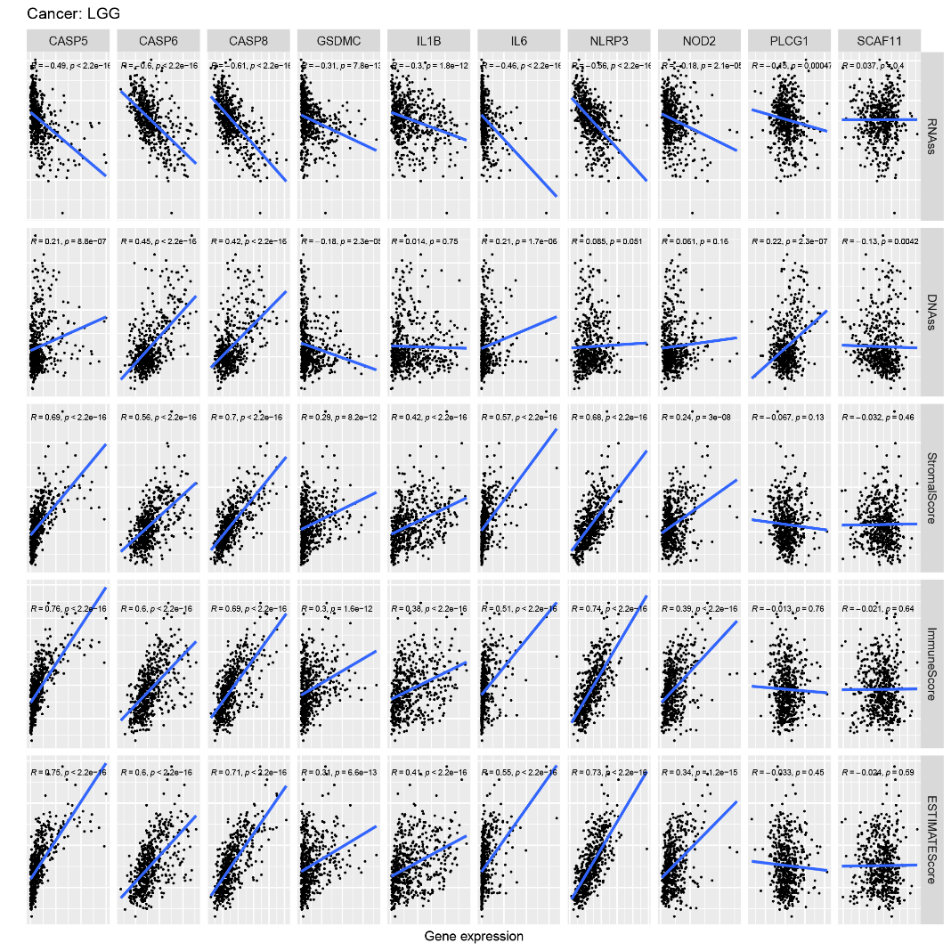
**

**Figure S9** Correlation of expressions of 10 signature genes with cancer stem cell-like properties (RNAss, DNAss) and TME (Stromal score, Immune score, and ESTIMATE Score. **(A)** RNAss. **(B)** DNAss. **(C)** Stromal score. **(D**) Immune score. **(E)** ESTIMATE score.

**
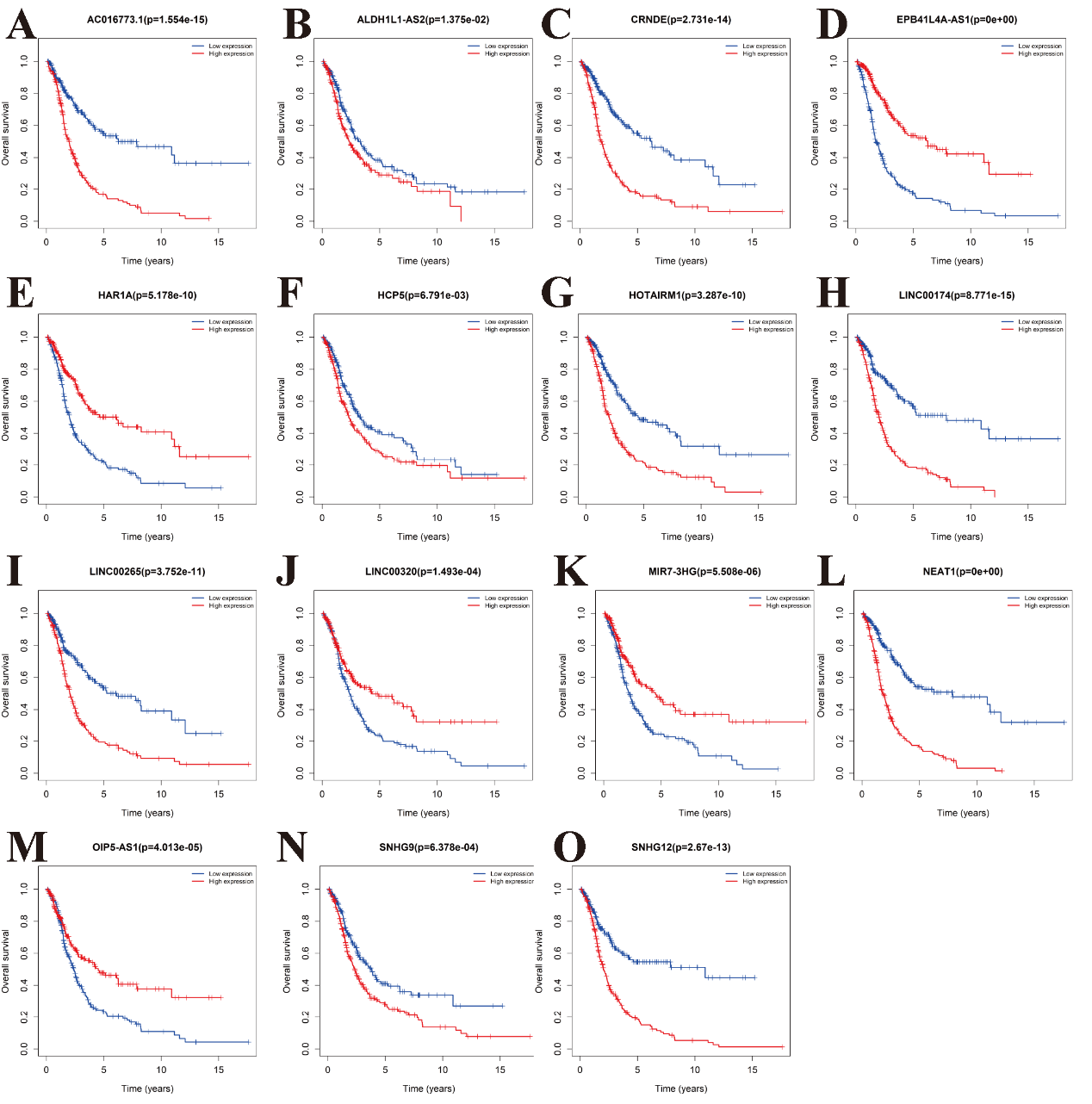
**

**Figure S10** Kaplan-Meier curves of lncRNAs for OS in the ceRNA network. **(A)** AC016773.1 **(B)** AlDH1L1-AS2. **(C)** CRNDE. **(D)** EPB41L4A-AS1. **(E)** HAR1A. **(F)** HCP5. **(G)** HOTAIRM1. **(H)** LINC00174 **(I)** LINC00266. **(J)** LINC00320. **(K)**MIR-3HG. **(L)**NEAT1. **(M)** OIP5-AS1. **(N)** SNHG9. **(O)** SNHG12.

**
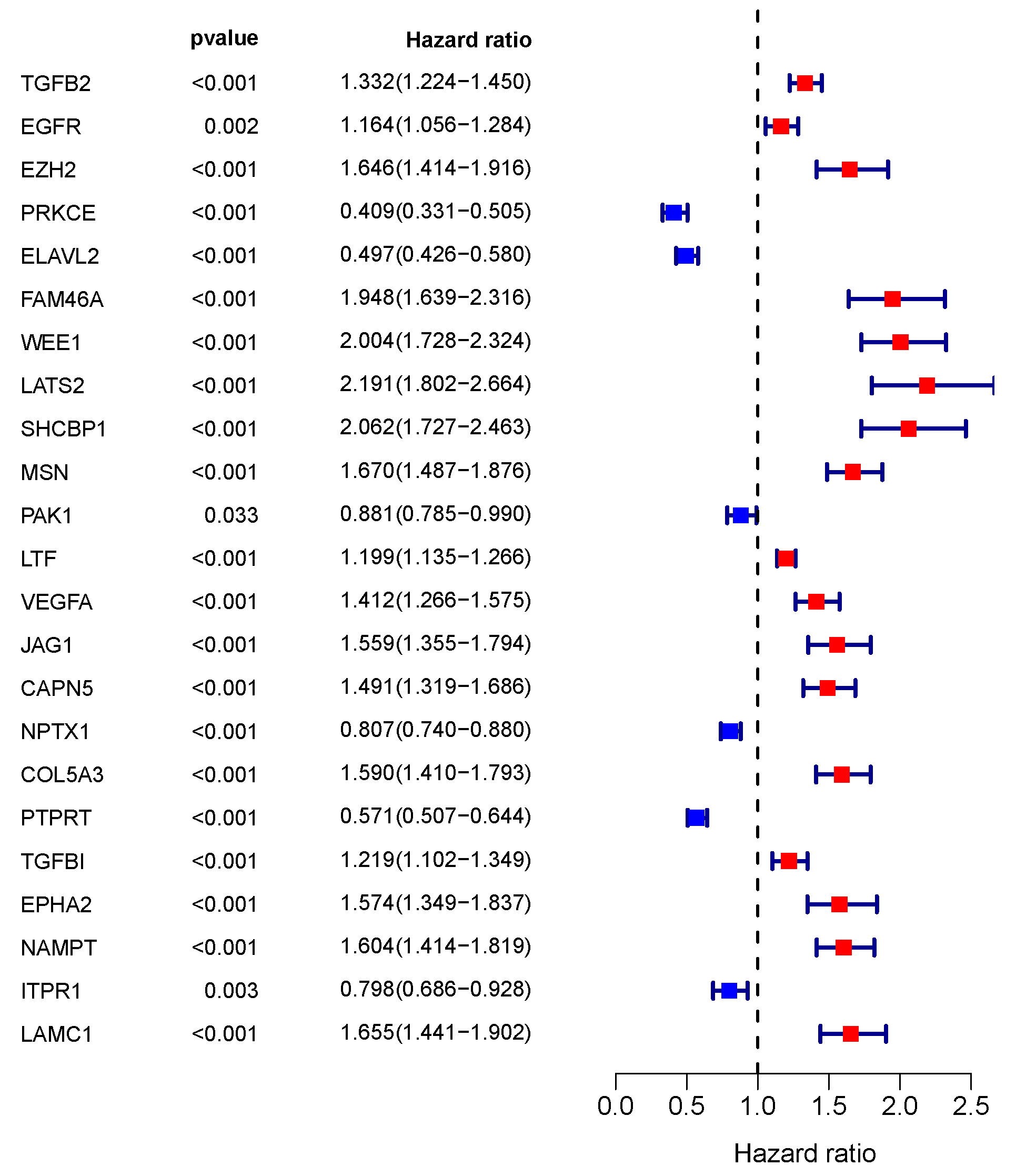
**

**Figure S11** Forest plot of 23 prognosis-related mRNAs in the ceRNA network

**
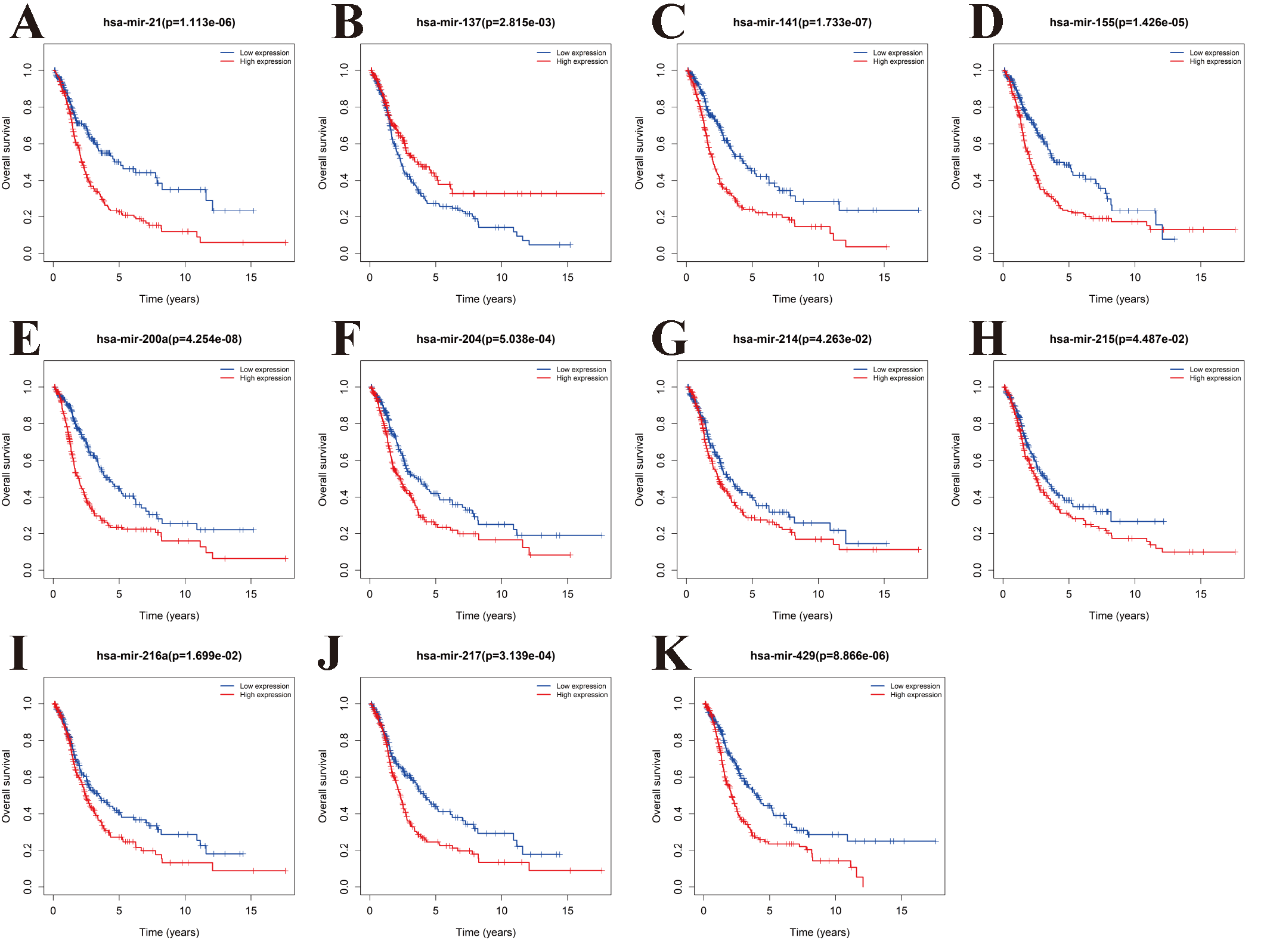
**

**Figure S12** Kaplan-Meier curves of mir-RNAs for OS in the ceRNA network. **(A)** mir-21. **(B)** mir-137. **(C)** mir-141. **(D)** mir-155. **(E)** mir-200. **(F)** mir-204. **(G)** mir-214. **(H)** mir-215. **(I)** mir-216a. **(J)** mir-217. **(K)** mir-429

**
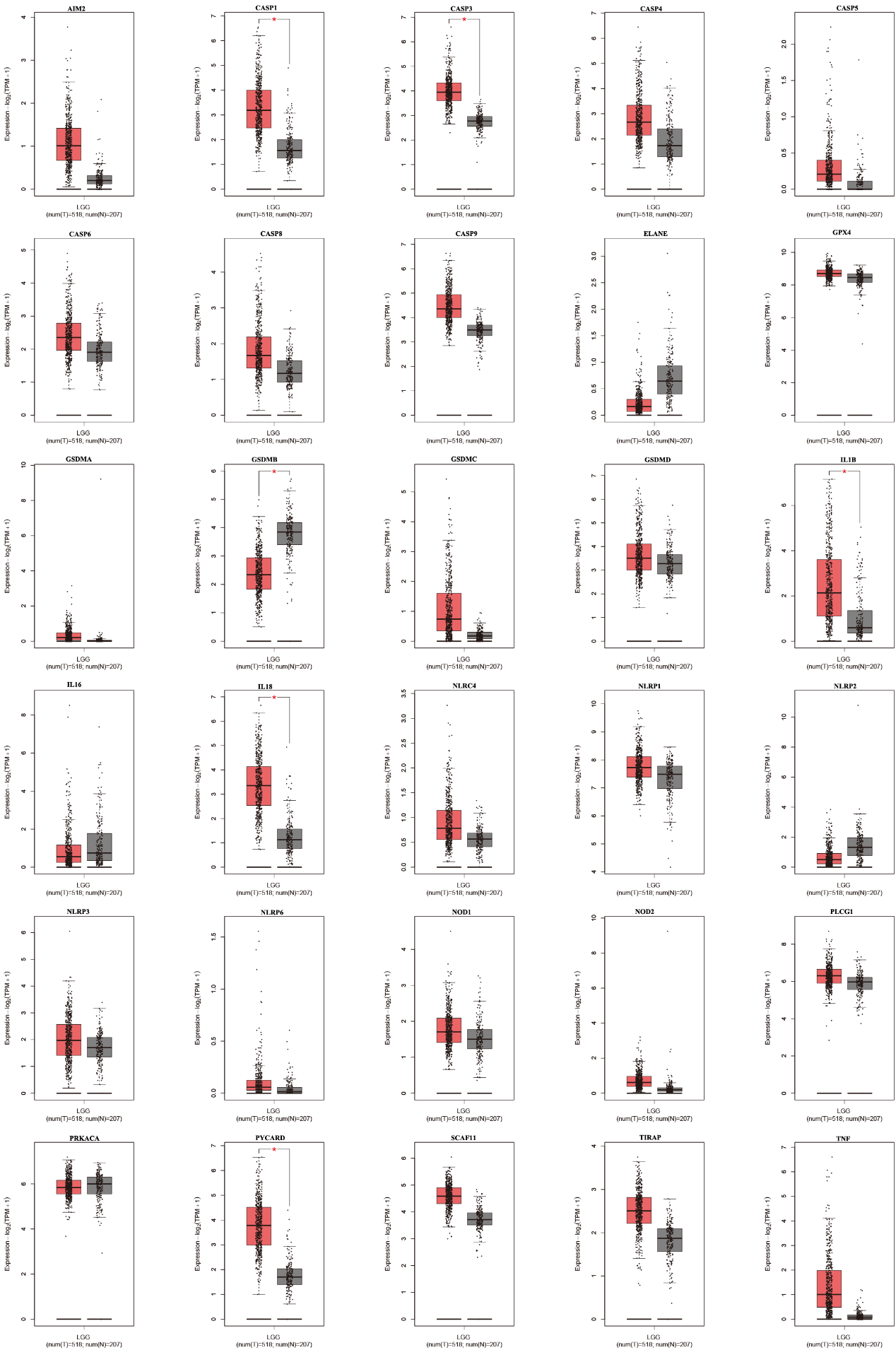
**

**Figure S13** The expression levels of pyroptosis genes between tumor (low-grade glioma) and normal (GTExdata)
