# Supplementary figures and images for: Integrative analysis reveals the functional implications and clinical relevance of pyroptosis in low-grade glioma
Source: Sci Rep. 2022 Mar 16;12:4527. doi: 10.1038/s41598-022-08619-w (PMC8925295; doi:10.1038/s41598-022-08619-w)

**Supplementary material 4** The original gels of Western blot for Figure 10A and Figure 10B


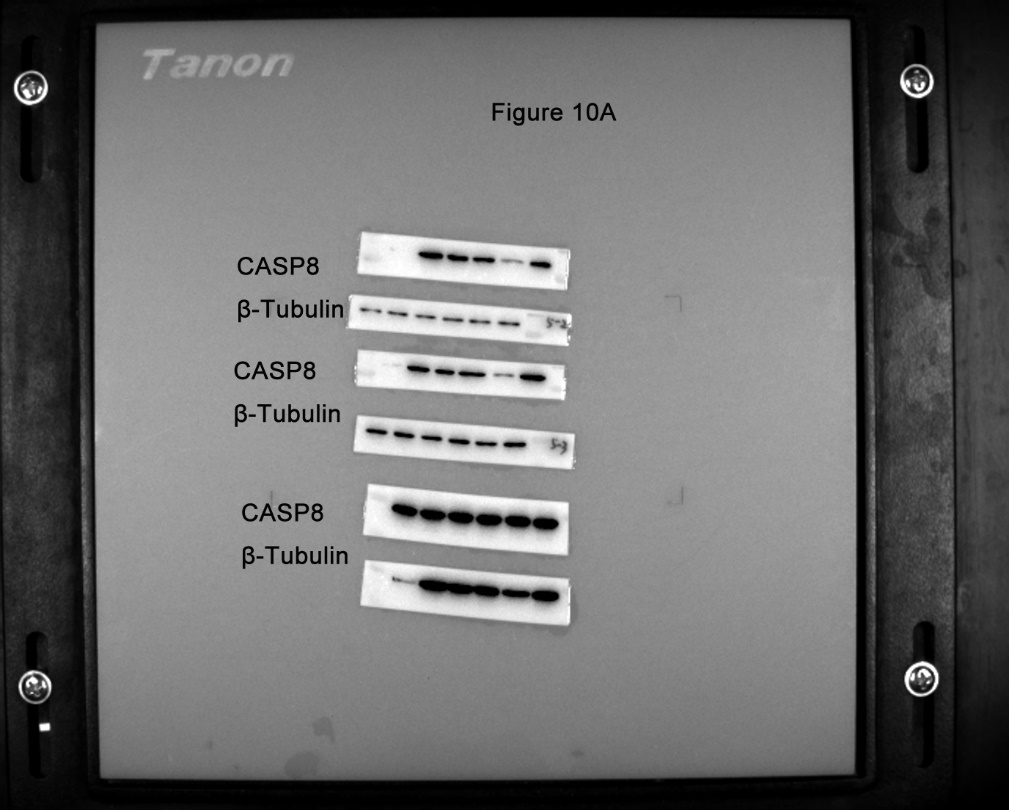


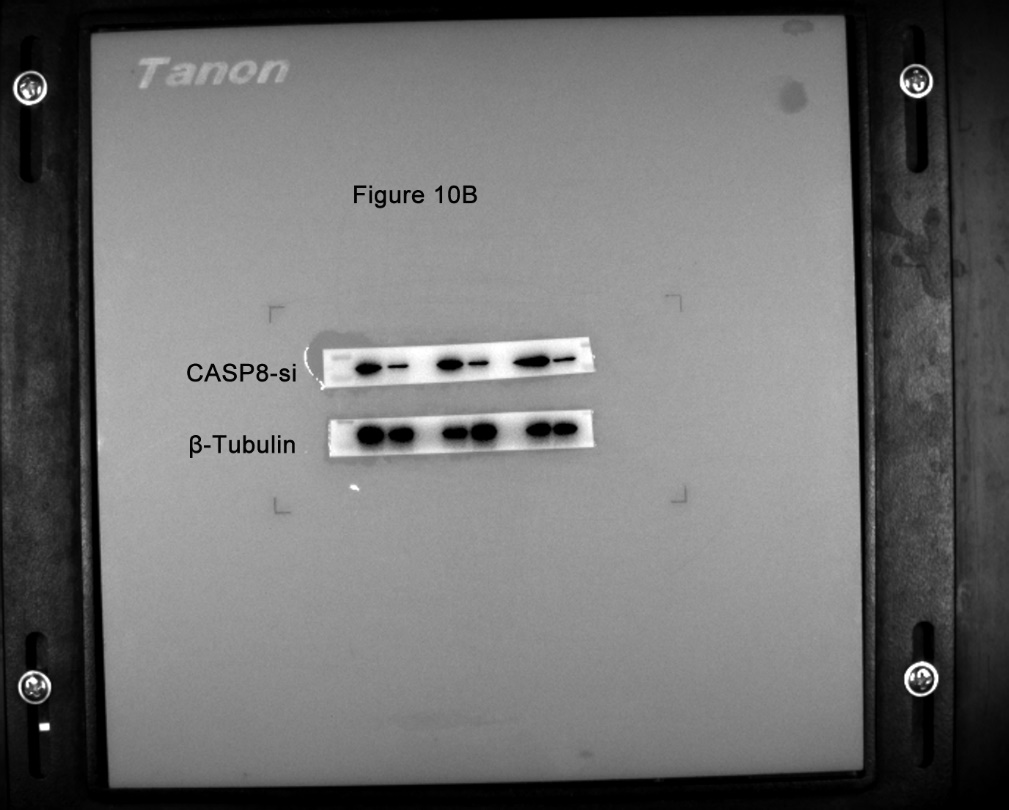

Supplement: Supplementary file 4 — Supplementary Information 4. [file 41598_2022_8619_MOESM4_ESM.docx]
